# Dynamic post-transcriptional regulation by Mrn1 links cell wall homeostasis to mitochondrial structure and function

**DOI:** 10.1371/journal.pgen.1009521

**Published:** 2021-04-15

**Authors:** Kendra Reynaud, Molly Brothers, Michael Ly, Nicholas T. Ingolia

**Affiliations:** 1 California Institute for Quantitative Biosciences, University of California, Berkeley, Berkeley, California, United States of America; 2 Department of Molecular and Cell Biology, University of California, Berkeley, Berkeley, California, United States of America; Ohio State University, UNITED STATES

## Abstract

The RNA-binding protein Mrn1 in *Saccharomyces cerevisiae* targets over 300 messenger RNAs, including many involved in cell wall biogenesis. The impact of Mrn1 on these target transcripts is not known, however, nor is the cellular role for this regulation. We have shown that Mrn1 represses target mRNAs through the action of its disordered, asparagine-rich amino-terminus. Its endogenous targets include the paralogous SUN domain proteins Nca3 and Uth1, which affect mitochondrial and cell wall structure and function. While loss of *MRN1* has no effect on fermentative growth, we found that *mrn1Δ* yeast adapt more quickly to respiratory conditions. These cells also have enlarged mitochondria in fermentative conditions, mediated in part by dysregulation of *NCA3*, and this may explain their faster switch to respiration. Our analyses indicated that Mrn1 acts as a hub for integrating cell wall integrity and mitochondrial biosynthesis in a carbon-source responsive manner.

## Author summary

Regulated degradation of messenger RNAs acts together with regulated transcription to determine mRNA abundance and thereby control gene expression. RNA-binding proteins coordinate post-transcriptional gene expression programs by recognizing groups of functionally related transcripts and modulating their stability and decay. Here, we establish that the yeast RNA-binding protein Mrn1 controls a gene expression program that links the mitochondrion with the yeast cell wall. The cell wall is a dynamic structure that withstands substantial osmotic pressure while undergoing continuous remodeling during growth and division. It comprises a large fraction of the cell’s dry weight and consumes substantial energy as well as carbohydrates. Mrn1 binds hundreds of target mRNAs, which are enriched for functions in cell wall organization and biogenesis. We found that Mrn1 destabilizes these targets during glucose fermentation, but its repressive effect is relieved when cells switch to respiratory growth. Cells lacking *MRN1* have expanded mitochondria during glucose fermentation and adapt more quickly to respiration. The SUN domain protein Nca3 is a key Mrn1 target that is associated with both the cell wall and the mitochondrion and underlies much of these effects. Mrn1 control of Nca3 and other genes sculpts mitochondria and the cell wall in response to changing growth conditions.

## Introduction

Gene expression is regulated post-transcriptionally by numerous proteins that recognize specific transcripts and modulate their translation, localization, and decay [1]. An individual RNA-binding protein (RBP) can target dozens or hundreds of different messenger RNAs (mRNAs), thereby controlling a post-transcriptional regulon of functionally related genes [2]. These coordinated regulatory programs allow cells to quickly remodel gene expression in response to changing growth conditions [3] and acute stress [4].

The yeast protein Mrn1 seems well suited to control such a post-transcriptional regulon. It contains four RNA-recognition motif (RRM) domains, giving it a substantial potential to recognize target transcripts specifically. Indeed, Mrn1 has been reported to bind over 300 mRNAs, and these targets are enriched for transcripts that encode cell wall biogenesis and regulatory proteins [5]. Many of these target transcripts are shared among a subset of other yeast RBPs including Khd1, Pub1, Scp160, and Ssd1 [5]. No overt cell wall phenotypes have been linked to the loss of *mrn1*, however, and prior work has instead identified genetic interactions with chromatin remodelers and splicing factors [6].

In addition to its four RRMs, Mrn1 contains an intrinsically disordered amino-terminal domain that begins with a low-complexity, poly-asparagine region. Disordered regions are common among RNA-binding proteins, and they can serve as interaction motifs that bind other proteins and recruit them to an mRNA [7]. Interactions between disordered regions also play important roles in the formation of phase separated ribonucleoprotein condensates, such as stress granules and P-bodies [8]. While Mrn1 appears diffusely cytosolic during normal growth, it localizes to P-bodies upon glucose deprivation [9]. Mrn1 is also hyper-phosphorylated under these conditions, similar to many other RBPs that localize to cytosolic granules in glucose starvation [10,11]. In contrast to these other proteins, however, Mrn1 hyper-phosphorylation was not relieved by the deletion of the kinase *SNF1*. This result suggests that Mrn1 may play a distinctive role in response to glucose withdrawal. Mrn1 may also respond to heat stress, as it was strongly depleted from mRNAs after heat shock [12].

Here, we showed that Mrn1 promotes turnover of its target transcripts, acting through the general mRNA decay factor *LSM3*. While many Mrn1 targets are indeed cell wall proteins, we also identified genetic and physical connections between Mrn1 and mitochondria. Loss of *MRN1* leads to mitochondrial expansion in fermentative conditions and a faster transition to respiratory growth. These effects appear to be mediated in part by *NCA3*, a strongly responsive Mrn1 target that regulates the mitochondrial ATPase [13] and is linked to cell wall biogenesis [14].

## Results

### The disordered N-terminus of Mrn1 promotes mRNA turnover

**Fig 1:**
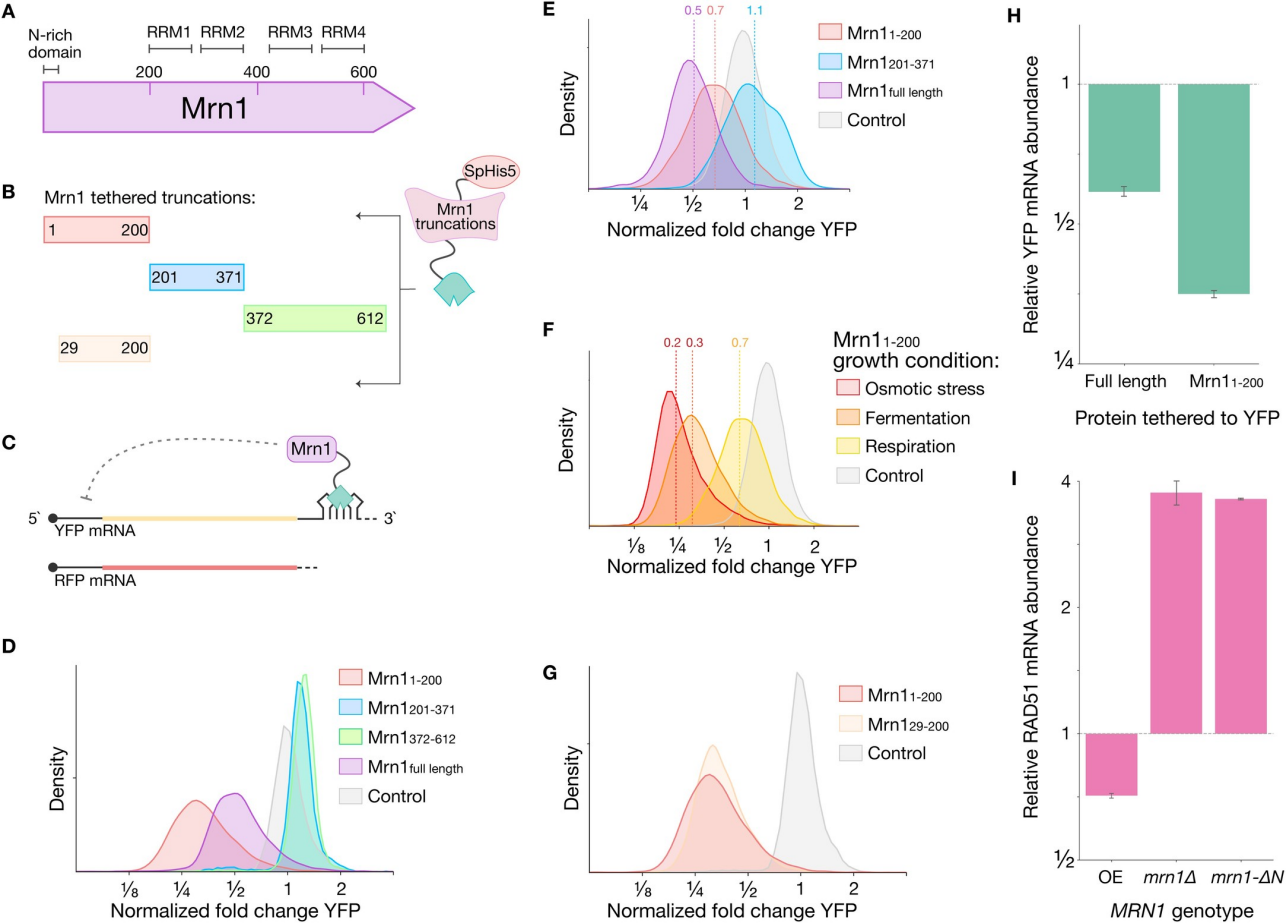
Mrn1 is a post-transcriptional repressor that stimulates mRNA degradation through its disordered N-terminus. (A) Predicted domain structure of Mrn1. (B) Mrn1 truncations tethered to mRNA reporter in tethering assay. (C) Schematic of tethering assay with YFP reporter and RFP normalization control. (D) Flow cytometry measuring activity of Mrn1 and its truncations in the tethering assay during fermentative growth and (E) respiratory growth (n = 50,000 cells per sample, one representative replicate shown in each histogram). (F) As in (D), for Mrn1(1–200) activity in the tethering assay during osmotic stress, fermentation and respiration. (G) As in (D), for Mrn1(1–200) and Mrn1(29–200) activity in the tethering assay during fermentative growth. (H) RT-qPCR analysis of YFP mRNA with indicated proteins tethered to the 3`UTR. Expression reported relative to a non-regulator control and normalized to an untargeted RFP mRNA. (I) RT-qPCR analysis of endogenous Mrn1 target *RAD51* expression normalized to the *UBC6* housekeeping gene. Error bars reflect standard deviation.

To learn how Mrn1 affects its target transcripts, and determine which regions of the protein are responsible for its regulatory effects, we subdivided Mrn1 into separate domains and characterized each using a tethered function assay [15]. Mrn1 contains an unstructured N-terminus followed by four predicted RNA-recognition motifs (RRMs) (Fig 1A). We split Mrn1 into three fragments, one comprising the disordered N-terminus, Mrn1(1–200), another the first two RRMs, Mrn1(201–371), and a third containing the third and fourth RRMs, Mrn1(372–612) (Fig 1B). The regulatory activity of each fragment was quantified by tethering it to the 3′ UTR of a yellow fluorescent protein (YFP) reporter mRNA and measuring YFP fluorescence relative to a red fluorescent protein (RFP) normalization reporter expressed in the same cells (Fig 1C). Mrn1(1–200) repressed reporter expression roughly 3.5-fold relative to an inactive control, whereas the full-length protein repressed the reporter only 2-fold (Figs 1D, S1A and S1B). Full-length Mrn1 may be subject to inhibition that Mrn1(1–200) evades by omitting the C-terminal regions of the protein. In contrast, Mrn1(201–371) and Mrn1(372–612) had little effect on normalized YFP (Fig 1D). While expression of these RRM fragments had no specific regulatory effect, we noted that total YFP and RFP fluorescence increased (S1C and S1D Fig), which we attributed to an increase in overall cell size (S1E Fig).

Because Mrn1 enters cytoplasmic ribonucleoprotein granules upon glucose deprivation [9], we were curious how its activity might change in different growth conditions. We repeated the tethering assay using cells grown in non-fermentable media with ethanol and glycerol as carbon sources. The repressive effect of N-terminal Mrn1(1–200) was substantially weaker in respiratory growth conditions, as reporter expression was reduced by only 30%, in contrast to the 70% reduction seen in fermentative growth on glucose. Full-length Mrn1 showed equally strong repression in these conditions, however, and the RRM-containing fragment Mrn1(201–371) retained its modest and largely non-specific effects (Fig 1E). Cells expressing the Mrn1(372–612) tethering fusion protein grew extremely slowly in the non-fermentable media, precluding measurement of its activity.

We next tested whether other stress conditions would also elicit a similar decrease in Mrn1(1–200) activity. Notably, Mrn1(1–200) actually became a stronger repressor following osmotic stress (0.6 M NaCl) (Fig 1F), consistent with the observation that Mrn1 binds transcripts involved in cell wall growth and expansion. For example, it has been shown that the loss of *UTH1*, previously identified as an Mrn1 target, leads to cells with more robust cell walls that are resistant to perturbations such as zymolyase and calcofluor white [5,16]. The increased repressive activity of Mrn1 during osmotic stress may produce a similar effect as deleting *UTH1* and thus create a more robust cell wall.

Mrn1 begins with a low-complexity region with 21 asparagine residues in the first 28 positions, including a stretch of 14 consecutive asparagines. Low-complexity domains are important in the formation of RNP granules as they allow RBPs to polymerize and undergo a reversible phase transition into a hydrogel-like state [17]. To assess the functional relevance of the low-complexity domain in Mrn1, we tested the activity of a tethering fusion lacking this amino-terminal asparagine-rich region. We saw minimal change in the activity of Mrn1(29–200), which lacked the poly-asparagine region, relative to Mrn1(1–200) (Fig 1G). Like Mrn1(1–200), the repressive effect of Mrn1(29–200) was also weaker in respiratory growth conditions (S1F Fig). However, osmotic stress did not enhance the repressive effect of Mrn1(29–200), indicating that the increase in Mrn1(1–200) activity during high-osmolarity is dependent on its low complexity sequence (S1G Fig).

The tethering assay provides an integrative measure of changes in translation and mRNA stability. In order to deconvolve these effects, we next assessed whether the regulatory effects of Mrn1 reflected translational repression or enhanced mRNA turnover. We measured reporter mRNA abundance by reverse transcription followed by quantitative PCR (RT-qPCR) in the presence of tethered full-length Mrn1 or N-terminal Mrn1(1–200) and compared this with tethering of an inactive Halo protein. We discovered that most of Mrn1’s activity is due to RNA turnover, as the decrease in mRNA abundance nearly matches the level of fluorescent protein repression we observed in the tethering assay (Fig 1H).

We wanted to test whether Mrn1 also promoted degradation of its endogenous target transcripts. The *RAD51* transcript was previously identified as one of the top endogenous targets of Mrn1 [5], so we expected that its abundance would increase upon *MRN1* deletion (*mrn1Δ*) and decrease when *MRN1* was over-expressed. Indeed, in *mrn1Δ* we observed RAD51 mRNA levels increase by approximately 4-fold, whereas over-expression of *MRN1* using the *P(PGK1)* promoter reduced *RAD51* expression nearly 2-fold. Finally, deletion of the genomic region spanning the first 200 amino acids in the *MRN1* locus (*mrn1-ΔN*) resulted in a 4-fold upregulation of RAD51(Fig 1I). These results confirm that Mrn1 is a post-transcriptional repressor that enhances the turnover of its endogenous mRNA targets through its disordered N-terminus.

### Mrn1-mediated RNA decay involves Lsm3

**Fig 2:**
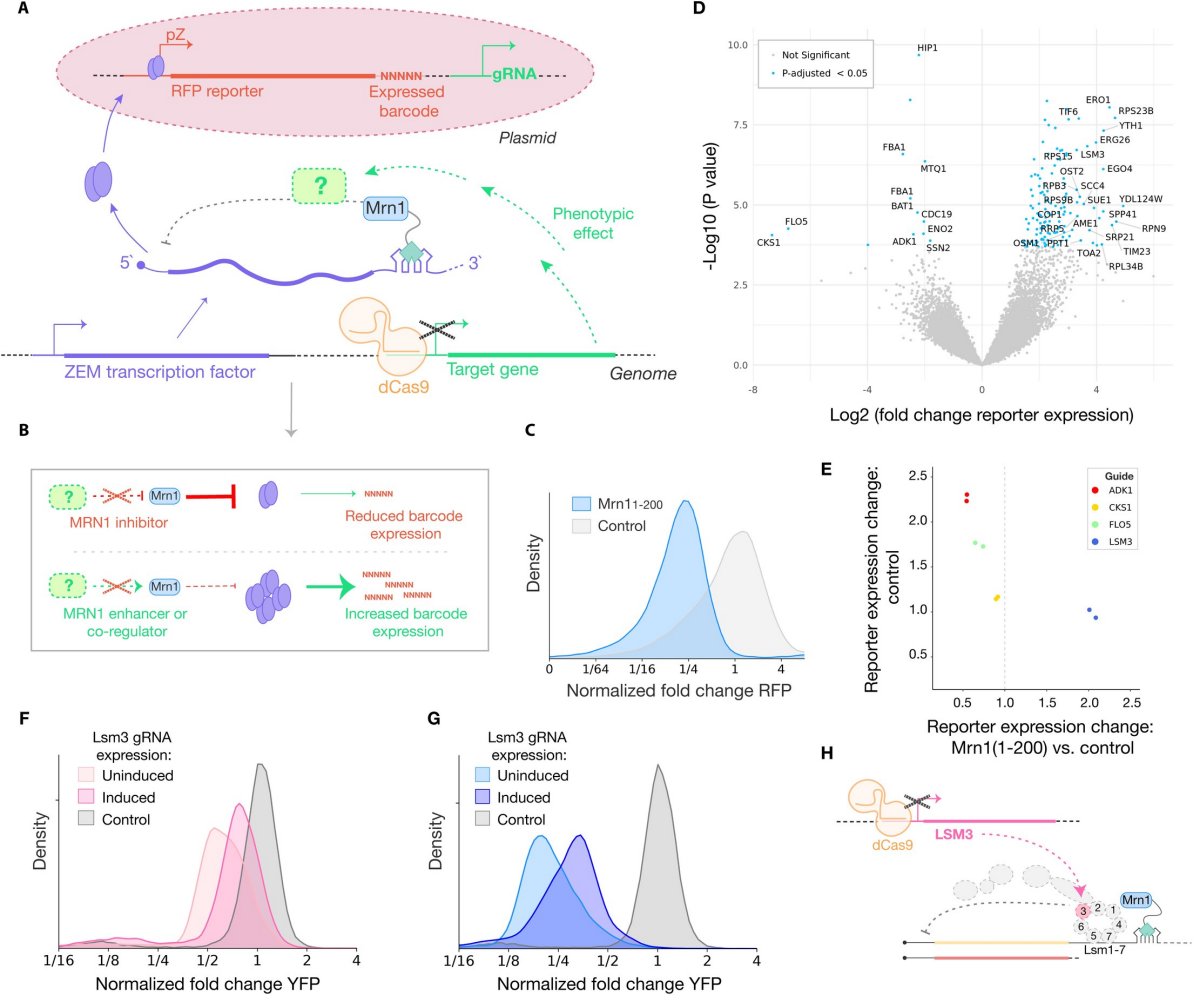
The tethering assay incorporated into CiBER-seq reveals Mrn1 RNA turnover mechanism involves Lsm3. (A) Schematic of indirect CiBER-Seq analysis of Mrn1 tethered to the mRNA encoding the ZEM transcription factor, which drives transcription of a barcoded RFP reporter. Target gene knockdown induced by dCas9-Mxi results in phenotypic changes in Mrn1 activity, quantitatively measured as changes in the expression of the RFP and barcode mRNA. (B) Knockdown of Mrn1 inhibitors results in stronger repression of reporter-barcode expression, whereas knockdown of Mrn1 co-repressors results in increased reporter-barcode expression. (C) Flow cytometry measurement of RFP expression with Mrn1(1–200), or an inactive control protein, tethered to 3`UTR of the ZEM transcription factor, normalized to untargeted YFP. Mrn1(1–200) activity is comparable to activity in (1D). (D) Indirect CiBER-Seq profile of Mrn1. Each point corresponds to one guide RNA. Labels correspond to significant genes with Log2 (change in expression) greater than three or less than zero. (E) Comparison of guide RNA effects on RFP expression with inactive control tether versus additional change in RFP expression with Mrn1(1–200) tethering. (F and G) Flow cytometry measurements of (F) full length Mrn1 activity and (G) Mrn1(1–200) activity with *LSM3* knockdown, relative to an inactive control tether and normalized to RFP. (H) Schematic of Mrn1 regulatory interaction with Lsm1p complex, mediated through Lsm3, and the impact of *LSM3* knock-down.

Having characterized Mrn1 as an RBP that destabilizes its targets, we next sought to identify the genetic requirements for its repressive activity. We recently developed an approach for CRISPR-based screening, called CiBER-Seq, in which expressed nucleotide barcodes linked uniquely with each guide RNA are quantified by deep sequencing in lieu of measuring expression changes using fluorescent reporter protein [18]. We linked CiBER-Seq with the tethered function assay in order to measure how CRISPRi-mediated genetic perturbations [19,20] changed the effect of Mrn1. Rather than directly tethering Mrn1 to the barcoded reporter transcript, we used an indirect approach wherein Mrn1 was tethered to an mRNA encoding a synthetic transcription factor, ZEM, which in turn regulated the expression of the barcoded reporter [21,22]. Genetic perturbations that alter the regulatory effect of Mrn1 should relieve or exacerbate Mrn1 repression of ZEM translational output, thereby impacting the expression of the downstream ZEM-driven barcoded reporter (Fig 2A). Each barcoded reporter is linked uniquely with one guide in a genome-scale library of tetracycline-inducible CRISPR interference (CRISPRi) guide RNAs [21]. We transformed this library of guide/reporter pairs into cells expressing the catalytically inactive CRISPRi effector protein dCas9-Mxi, which silences target gene expression, along with the Mrn1 tethering fusion and the ZEM tethering target.

Prior to gRNA induction, Mrn1 should repress expression of *ZEM*, just as it repressed expression of the fluorescent reporter. Upon gRNA induction, knockdown of genes that promote Mrn1 activity or serve as co-repressors would relieve Mrn1-mediated repression of ZEM and thereby increase expression of the barcoded reporter. Likewise, knockdown of genes that typically inhibit Mrn1 should enhance its repressive effect (Fig 2B). We verified that the repressive effects of Mrn1(1–200) tethered to the *ZEM* 3′ UTR propagated through to affect the downstream reporter transcript and elicited a ~4-fold reduction in expression (Fig 2C). We also verified that this effect was general by tethering Pat1, a protein that activates RNA decapping and deadenylation, to the 3`UTR of the ZEM transcript (S2A–S2C Fig) [23,24].

We carried out CiBER-Seq analysis of Mrn1(1–200) tethering and compared these results with tethering of an inactive control, the Halo-tag protein, in order to identify Mrn1-specific effects (Fig 2D). Mrn1 activity increased upon knockdown of a range of genes involved in cell cycle regulation, cell wall biogenesis, mitochondrial respiration, and glycolysis. These regulatory effects suggest a potential negative feedback loop that decreases Mrn1 activity in certain cell cycle stages and in response to stresses such as the switch to respiration. We also identified CRISPRi targets where genetic perturbations decreased Mrn1 activity, including *LSM3*, *LSM4*, and *LSM5*, three subunits of the heptameric Lsm1 complex involved in cytoplasmic mRNA turnover [25]. *LSM3* knockdown resulted in the strongest and most significant change in Mrn1 activity among the subunits of the Lsm1 complex. Other factors involved in mRNA degradation included the 5’-to-3’ exonuclease *XRN1*, the ATP-dependent RNA helicase *DBP2*, and *EAP1*, which accelerates decapping [26–28].

We selected a handful of genes identified in our CiBER-Seq screen, including *LSM3*, for targeted validation. We performed CRISPRi knockdown, as in the CiBER-Seq screen, and measured changes in the expression of the RFP reporter via flow cytometry rather than barcode sequencing. Knockdown of *LSM3* increased reporter expression 2-fold when Mrn1 was tethered to the ZEM transcript, but had no effect when Halo was tethered, suggesting that the Lsm1 complex, and Lsm3 in particular, may mediate Mrn1 repressive effects. In contrast, knockdown of *ADK1*, *FLO5*, or *CKS1* caused a relative decrease in reporter expression in Mrn1 tethering relative to Halo, principally because Mrn1 tethering blocked the positive effect that arose in the control (Fig 2E). These effects were all CRISPRi-dependent, because no guide appeared to affect reporter expression prior to guide induction (S2D Fig).

In order to further validate the importance of *LSM3* for Mrn1-mediated repression, we inhibited *LSM3* in the context of Mrn1 tethered directly to a fluorescent reporter transcript. Knock-down of *LSM3* reduced the repressive effects of full-length Mrn1 (Fig 2F) as well as the intrinsically disordered N-terminal Mrn1(1–200) (Fig 2G), confirming that Lsm3 activity is an integral part of the Mrn1 mechanism of mRNA turnover (Fig 2H).

### Mrn1 regulates cell wall biogenesis and organization transcripts

**Fig 3:**
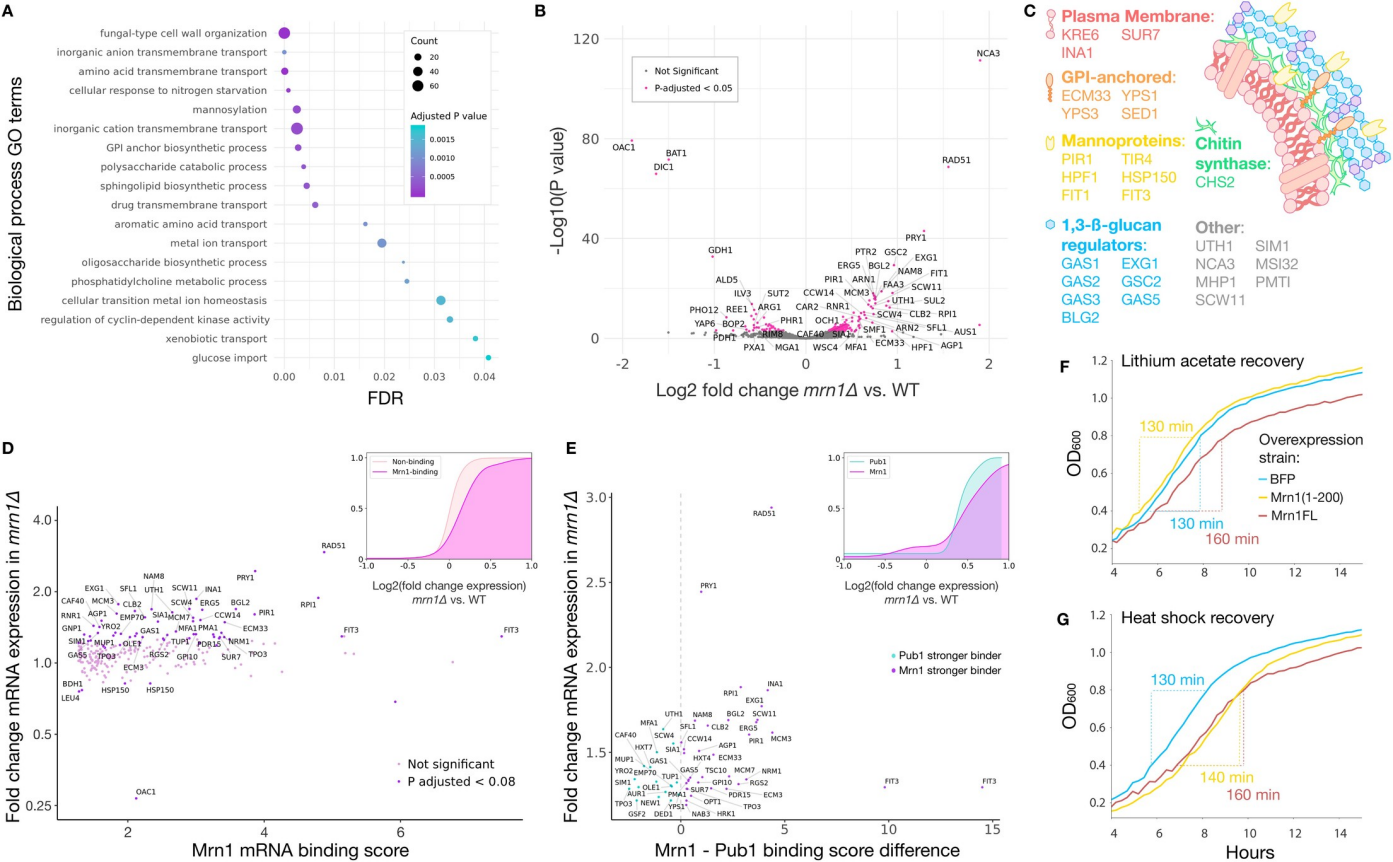
Mrn1 regulates turnover of cell wall organization and biogenesis RNAs. (A) Biological process GO terms enriched in mRNAs bound by Mrn1. (B) Comparison of the transcriptome in *mrn1Δ* versus wild-type yeast during fermentative growth (n = 3). Labels correspond to significant genes with Log2 (change in expression) greater than 0.5 or less than -0.5. (C) Schematic of cell wall organization and biogenesis genes upregulated in *mrn1Δ*. (D) Comparison of Mrn1 binding with expression difference in *mrn1Δ*. (inset) Cumulative distribution of expression differences in *mrn1Δ* yeast, stratified by Mrn1 binding status. (E) Comparison of RBP mRNA binding score with expression difference in *mrn1Δ* [5]. (inset) Cumulative distribution of expression differences in *mrn1Δ* yeast, stratified by Pub1 and Mrn1 binding. (F and G) Growth of strains over-expressing BFP, Mrn1, or Mrn1(1–200) after (F) lithium acetate stress and (G) heat shock stress. Time is reported for hours of growth after stress, (n = 3, one representative sample per strain is depicted).

Mrn1 associates with over 300 mRNAs, and dozens of these Mrn1 targets encode proteins that localize to the cell wall, plasma membrane, or extracellular matrix [5]. We performed gene ontology (GO) term enrichment analysis on the list of Mrn1 targets to gain a more complete picture of the processes it regulates. This analysis revealed that, in addition to cell wall homeostasis, Mrn1 targets were also enriched in functional annotations related to transmembrane transport, including glucose import, and cyclin-dependent kinase activity (Fig 3A).

To further explore the impact of Mrn1-mediated regulation on gene expression, we carried out RNA sequencing in wild-type and *mrn1Δ* cells. Because Mrn1 destabilizes its target mRNAs, we expected that *mrn1Δ* would exhibit an upregulation of these transcripts. We grew triplicate cultures of *mrn1Δ* and wild-type yeast in rich, glucose-replete media, harvested exponentially-growing cells, and performed RNA-seq. Indeed, we found significantly higher levels of over 50 RNAs in *mrn1Δ* cells that were previously identified as Mrn1 targets [5], including over a dozen involved in cell wall homeostasis (Fig 3B). The upregulated transcripts encode proteins involved in 1,3-β-glucan synthesis and regulation as well as mannoproteins, GPI-anchored proteins, chitin synthases and plasma membrane proteins that regulate the cell wall (Fig 3C). In fact, gene ontology enrichment analysis indicated that over 30 of the mRNAs upregulated in *mrn1Δ* are classified under fungal-type cell wall organization, a significant over-representation of this annotation (*q* = 0.04, hypergeometric test). Although cell wall regulatory RNAs were most numerous among those that were upregulated in *mrn1Δ*, we noted that the individual RNAs showing the strongest expression changes were involved in mitochondrial organization and biosynthesis, including *NCA3*, *OAC1*, *BAT1*, and *DIC1*. Notably, the *NCA3* paralog *UTH1* was also upregulated. This gene encodes a protein showing dual localization to the cell wall and the mitochondrial membrane [13] whose deletion leads to a more robust cell wall [16]. More broadly, the expression changes seen in *mrn1Δ* suggests that Mrn1 links these two organelles.

In order to differentiate between direct effects on Mrn1 targets versus indirect effects on downstream mRNAs, we constructed an Mrn1-mRNA binding score and compared this score with the relative change in expression caused by *mrn1Δ*. We derived this binding score from the Mrn1 enrichment reported by Hogan, *et al*. for all transcripts found to have a statistically significant interaction with Mrn1 (Fig 3D). We found that the RNAs with significant evidence for an interaction with Mrn1 tended to have a larger expression change in *mrn1Δ* (Fig 3D inset), indicating that genetically perturbing *MRN1* broadly results in the upregulation of its target mRNAs. However, we noted that this trend was not universal, and in some cases the change in RNA abundance of Mrn1 targets was quite mild. Hogan *et al*. noted that Mrn1 had a very similar list of targets as Pub1, a poly(A)-binding protein that is important for the stability and translation of over 500 transcripts [29,30]. In order to investigate whether Pub1 binding mitigated the impact of Mrn1 regulation, we first identified a set of likely target mRNAs that bound Mrn1 and showed higher abundance in *mrn1Δ* relative to wild-type. We then subdivided these transcripts based on whether they bound Mrn1 better than Pub1 or vice versa, based on the difference in their Mrn1 versus Pub1 binding scores. In this analysis, transcripts that bound Pub1 better than Mrn1 tended to have a weaker change in expression in *mrn1Δ*, whereas those that bound Mrn1 better had a stronger change in expression (Wilcoxon rank-sum test, *p* = 0.016) (Fig 3E). This suggests a competitive interaction between Pub1 and Mrn1 in regulating shared targets, whereby those transcripts that are more tightly bound by Pub1 are more protected from Mrn1-mediated degradation. In support of this theory, Hogan *et al*. reported very similar RNA sequence motifs for Mrn1 and Pub1 binding [5].

Finally, since *MRN1* represses transcripts involved in cell wall organization and biogenesis, we reasoned that *MRN1* over-expression would hinder the cell’s recovery from stresses impacting the cell wall. The cell wall integrity pathway is induced in response to prolonged elevated temperatures above 37°C [31], and Mrn1 demonstrated the strongest reduction in RNA-binding out of all proteins in yeast stressed at 42°C for 16 minutes [32]. Lithium acetate, routinely used in yeast transformation, also acts to perturb the cell wall [33]. We subjected strains over-expressing full-length or N-terminal *MRN1*, along with a BFP over-expression control, to two forms of cell wall stress: a 20-minute heat shock at 42°C, and treatment with 100 mM lithium acetate. After lithium acetate stress, cells expressing the N-terminal *MRN1* fragment recovered from stress as rapidly as the BFP control strain and had the same doubling time. However, cells overexpressing full-length *MRN1* were slower to recover and had a longer doubling time post-stress (Fig 3F). Both full-length and N-terminal *MRN1* overexpression strains took longer to resume growth after heat shock than the control, though N-terminal *MRN1* overexpression returned to a nearly normal growth rate (Fig 3G). The effect of overexpressing the N-terminal fragment, which is not expected to bind endogenous target mRNAs, may be explained by the added stress of over-expressing a low-complexity disordered domain, since heat shock triggers protein aggregation and misfolding [34]. Interestingly, *MRN1* overexpression does not have a universal deleterious impact on the cell’s response to cell wall stressors. We subjected the BFP and *MRN1* overexpression strains, as well as *mrn1Δ*, to osmotic stress in 0.6 M NaCl. We discovered that the *MRN1* overexpressing cells recovered more quickly and grew at a faster initial growth rate after high salt stress, whereas *mrn1Δ* exhibited virtually no difference in growth when compared to the BFP overexpression control (S3H Fig). This phenotype is consistent with the observation that Mrn1(1–200) repression is stronger in high salt media. These results also suggest that Mrn1’s role in the cell’s response to cell wall stress is dynamic and depends on the type of stress introduced.

### Mrn1 represses expression of mitochondrial mRNAs during fermentative growth

**Fig 4:**
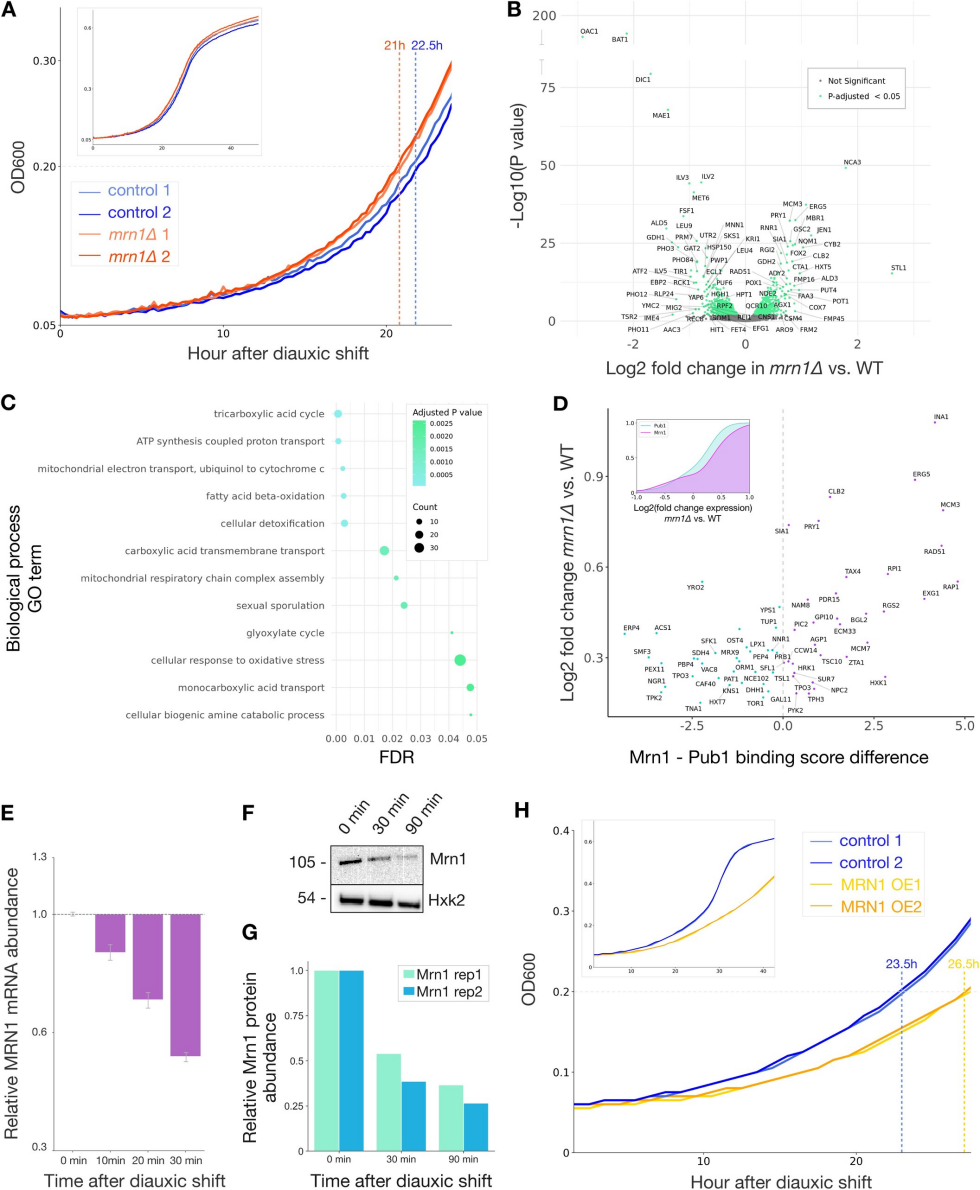
Mrn1 represses mitochondrial adaptation to respiration during fermentative growth. (A) Growth of *mrn1Δ* and wild-type yeast after the switch from fermentative to respiratory growth indicates a faster adaptation to non-fermentable media in *mrn1Δ* cells (n = 3, two representative replicates per strain depicted in growth curve). (B) Comparison of the transcriptome in *mrn1Δ* versus wild-type yeast 20 minutes after shift to non-fermentable media (n = 3). Labels correspond to significant genes with Log2 (change in expression) greater than 0.6 or less than -0.6. (C) Gene ontology analysis of mRNAs upregulated in *mrn1Δ* versus wild-type during respiratory growth. (D) Comparison of RBP mRNA binding score with expression difference in *mrn1Δ* during respiratory growth [5]. (inset) Cumulative distribution of expression differences in *mrn1Δ* yeast during respiratory growth, stratified by Pub1 and Mrn1 binding. (E) RT-qPCR measurement of *MRN1* expression over a timecourse after changing from glucose to non-fermentable ethanol and glycerol (n = 3). Measurements relative to *MRN1* abundance at zero minutes post-shift. Error bars reflect standard deviation. (F) Immunoblotting of Halo-tagged Mrn1 protein and Hxk2 control protein over a timecourse after changing from glucose to non-fermentable ethanol and glycerol (n = 2). (G) Quantification of (F). (H) Growth of cells over-expressing Mrn1 after the switch from fermentative to respiratory growth (n = 3, two representative replicates per strain depicted).

Mrn1 localizes to cytoplasmic granules in response to glucose starvation [9]. Glucose depletion also de-represses the use of other carbon sources and triggers cellular transition from aerobic fermentation to respiration. Mitochondria play a central role in respiratory growth and they expand and change substantially during the diauxic shift from glucose fermentation to ethanol utilization [35]. Our data show that *MRN1* affects the expression of dozens of genes with mitochondrial functions, and the repressive effect of Mrn1 is weaker during respiratory growth. These results suggested that *MRN1* may play a role in the physiological changes occurring during the diauxic shift. To test this more directly, we grew *mrn1Δ* and wild-type yeast in fermentable glucose and switched these cultures to non-fermentable media with glycerol and ethanol as the only carbon sources. Interestingly, *mrn1Δ* cultures returned to growth more quickly, entering exponential growth phase almost one full doubling-time sooner than wild-type cells (Fig 4A).

The accelerated respiratory shift in *mrn1Δ* cells led us to profile mRNA abundance changes that occur during this metabolic reprogramming process. We hypothesized that by comparing the early changes in transcript abundance during the shift from aerobic fermentation to respiration we would learn the basis for the accelerated recovery of *mrn1Δ* relative to wild-type cells. Indeed, RNA-seq of triplicate wild-type and *mrn1Δ* cultures 20 minutes after a shift from glucose to non-fermentable ethanol and glycerol revealed that over 60 of the transcripts upregulated in *mrn1Δ* were involved in some stage of the mitochondrial response to respiration. *NCA3* was again one of the most highly upregulated transcripts in *mrn1Δ* relative to wild-type cells, although in both genotypes it was downregulated around 4-fold relative to fermentative conditions. Other transcripts upregulated at least 2-fold in *mrn1Δ* relative to wild-type included genes whose expression are typically repressed during growth on glucose, including the glycerol symporter *STL1*, the lactate transporter *JEN1*, the cytochrome *CYB2*, and the hexose transporter *HXT5* (Fig 4B). Gene ontology enrichment analysis of the mRNAs upregulated in *mrn1Δ* relative to wild-type identified significant terms linked to respiration, including the tricarboxylic acid cycle, the mitochondrial electron transport chain, and the glyoxylate cycle, among others (Fig 4C). Our analysis indicates that Mrn1 suppresses expression of these genes during fermentative growth and in the early stages of diauxic shift. One possibility is that Mrn1 restrains mitochondrial size in a manner that is beneficial for fermentative growth but slows the adaptation to respiration.

We noted the correlation between mRNAs that were more highly upregulated in *mrn1Δ* and those that preferentially bind Mrn1 over Pub1 was stronger after the shift to non-fermentable carbon sources than during fermentation (Fig 4D) (Wilcoxon rank-sum test, *p* = 7.5e-4). This may indicate competition for mRNA targets between Mrn1 and Pub1 serves as an initial point of triage for transcripts destined to be turned over or stored in stress granules during the early stages of diauxic shift [36].

We also quantified the expression of *MRN1* itself during the shift to respiratory growth. Levels of endogenous *MRN1* mRNA declined by roughly 50% over the 30-minute period following a shift from glucose to a non-fermentable carbon source (Fig 4E). In order to track how this transcript change affected Mrn1 protein, we introduced a Halo-tag into the endogenous *MRN1* locus and monitoring its abundance by immunoblotting (Fig 4F). The level of Mrn1 protein decreased by roughly 50% in 30 minutes following a shift to respiratory growth, similar to the reduction we saw in mRNA levels, and after 90 minutes it had decreased to about 30% of fermentative levels (Fig 4F). These results indicate that, as soon as diauxic shift begins, the cell represses Mrn1 expression, relieving the downregulation of mitochondrial gene expression.

Since *MRN1* overexpression interfered with cell wall stress responses, we were curious whether it would likewise interfere with cell growth during the shift to respiration. We switched wild-type and *MRN1* overexpression yeast from dextrose to a non-fermentable carbon source and monitored cell growth. We noticed that, after an extended lag phase, wild-type yeast resumed growth sooner than those overexpressing *MRN1* and maintained a faster rate of growth throughout the entire experiment (Fig 4H). We hypothesize that this growth phenotype again stems from the enhanced turnover of Mrn1 targets in the overexpression strain, which interferes with proper adaptation to respiratory growth.

To learn more about Mrn1 co-regulators, we investigated the proteins that interact with Mrn1 in normal and low glucose conditions. We inserted a Halo tag with a TEV-cleavable linker into the endogenous *MRN1* gene. As a control, we integrated mCherry with the same tag in a safe locus in the genome [37] (S4G and S4H Fig). We captured in vivo interaction patterns for these proteins by ex vivo crosslinking in lysates prepared by cryogenic pulverization followed by covalent capture of each query protein through the Halo fusion, along with crosslinked interactors. After stringent washing of these covalently coupled complexes, we eluted proteins by TEV cleavage and analyzed the captured interactors by quantitative mass spectrometry. We found a strong quantitative correlation (*r* = 0.75) between the Mrn1-to-mCherry peptide abundance ratios in lysates prepared from cells in rich or low-glucose media, arguing that our strategy to survey Mrn1 interactions was robust (S4I Fig). Gene ontology enrichment analysis of these interacting proteins identified biological processes including gluconeogenesis and glycolysis, translation termination, redox homeostasis, and protein folding (S4J Fig). Enriched cellular component terms include cytoplasmic stress granules, consistent with the known localization of Mrn1, as well as the polysome and the translation preinitiation complex, which enter cytoplasmic granules during glucose deprivation (S1 Table).

The Mrn1-interacting proteins we identified support connections between Mrn1 and the mitochondrion as well as the cell wall (S4I Fig and S1 Table). Indeed, Mrn1 crosslinked with several cell wall regulatory proteins that also have some link to the mitochondria (S1 Table, bold). For example, Pir1 is required for cell wall stability and also mediates mitochondrial translocation of Apn1, which is involved in maintaining mitochondrial genome integrity [38]. Ecm33 is involved in efficient glucose uptake and a phosphorylated form localizes to the mitochondria [35]. And finally, Zeo1 is a plasma membrane protein that regulates the cell wall integrity pathway and resides in a phosphorylated form in the mitochondria [39]. Several of the proteins most enriched for Mrn1 interaction, including Pir1, Ecm33, and other cell-wall-regulatory proteins, are encoded by mRNAs that are targeted by Mrn1 as well (S4K Fig). Together with our RNA-seq data, these findings support the hypothesis that Mrn1 coordinates the physically distinct cell wall and the mitochondria.

We also compared the genetic and physical interactions of Mrn1. In lysates from cells growing by respiration, Lsm1 was enriched over 2-fold in the Mrn1 co-purification relative to the mCherry control, although we did not detect any physical interactions between Lsm1-7 subunits and Mrn1 in fermentative cell lysate (S4L and S4M Fig). Greater interaction with Lsm1-7 in respiratory growth may be due in part to Mrn1 localization to P-bodies during glucose deprivation, as Lsm1 is a major coordinator of P-body formation [40,41].

### Mrn1 and its key target *NCA3* modulate mitochondrial size and function

**Fig 5:**
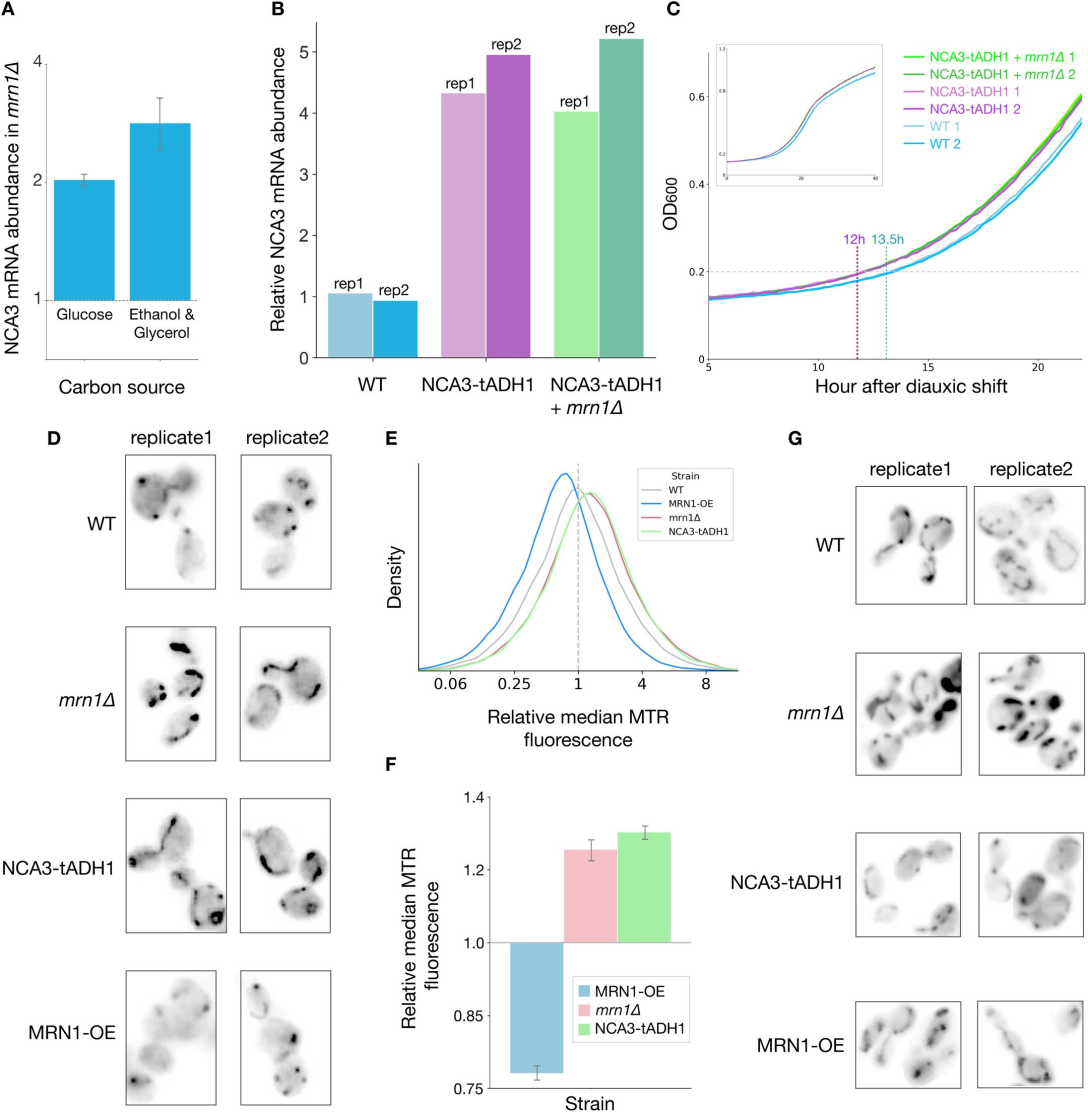
Mrn1 regulates mitochondrial expansion through control of *NCA3*. (A) RT-qPCR analysis of *NCA3* expression in *mrn1Δ* relative to wild-type cells. Error bars reflect standard deviation (n = 3). (B) As in (A), with replacement of the 3`UTR of endogenous *NCA3* with that of *ADH1* with wild-type *MRN1* (*NCA3-tADH1)* and with *mrn1Δ* (*NCA3-tADH1+ mrn1Δ*) (n = 2). (C) Growth of *NCA3-tADH1*, *NCA3-tADH1+ mrn1Δ* and wild-type cells after diauxic shift (n = 3, two representative replicates per strain depicted). (D) Visualization of mitochondria, stained by mitoTracker Red (MTR), in yeast undergoing fermentative growth. (E) Flow cytometric analysis of MTR fluorescence as a measure of mitochondrial abundance. (F) Quantification of median MTR fluorescence in (E), Error bars reflect standard deviation (n = 2). (G) As in (D) for yeast undergoing respiratory growth.

Oxidative phosphorylation uses the electrochemical gradient across the inner mitochondrial membrane to drive ATP synthesis by the multi-subunit F1-F₀ ATP synthase. Expression of mitochondrially-encoded subunits of the ATP synthase complex, Atp6p and Atp8p, is affected by *NCA3*, one of the most prominent Mrn1 target transcripts [13]. *NCA3* is a member of the SUN family of genes (*SIM1*, *UTH1*, *NCA3* and *SUN4)*, which are involved in diverse cellular processes, including the remodeling of the cell wall during various stages of growth and stress [42]. We found *NCA3* to be the most-highly upregulated transcript in fermentative conditions and the second mostly-highly upregulated transcript in respiratory conditions in *mrn1Δ* relative to wild-type cells (Figs 3B and 4B). We confirmed by RT-qPCR that *NCA3* is upregulated in *mrn1Δ* cells by approximately 2-fold during growth on glucose and 3-fold during growth on ethanol and glycerol, which closely matches the expression differences that we observed by RNA-seq, and conclude that Mrn1 activity strongly downregulates *NCA3* expression (Fig 5A).

While Hogan *et al*. identified *SIM1*, *UTH1*, and *SUN4* as targets of Mrn1, *NCA3* was not included in the study, and so it is unclear whether Mrn1 regulates *NCA3* directly. We noted, however, that the 3′ untranslated region (UTR) of *NCA3* contains three sites that match the Mrn1-binding motif [5], so it is quite plausible that Mrn1 binds *NCA3* as well. We replaced the 3′ UTR of the endogenous *NCA3* locus with that of *ADH1*, a widely-used, stabilizing 3′ UTR which contains no binding sites for Mrn1 and does not substantially change expression in our RNA-seq analysis of *mrn1Δ* yeast. The abundance of *NCA3-tADH1* was substantially higher than wild-type *NCA3* mRNA (Fig 5B), consistent with Mrn1-mediated down-regulation of *NCA3*, and furthermore, deletion of *MRN1* in *NCA3-tADH1* (*NCA3-tADH1* + *mrn1Δ*) resulted in no additional upregulation of *NCA3*. These results support a model wherein Mrn1 directly binds the 3′ UTR of *NCA3* and promotes its degradation, although we cannot exclude the possibility that 3′ UTR replacement interferes with the binding of other proteins that negatively regulate *NCA3* expression alongside Mrn1.

Since *NCA3-tADH1* should eliminate direct regulation of *NCA3* by Mrn1, we were curious whether this UTR substitution was sufficient to recapitulate the phenotypes we observed in *mrn1Δ*. Indeed, we noted that *NCA3-tADH1* resumed growth more quickly than WT after transfer to a non-fermentable carbon source (Fig 5C). Furthermore, the *NCA3-tADH1 mrn1Δ* double mutant also recovered more quickly and grew at a similar rate to *NCA3-tADH1*, indicating that the accelerated recovery seen in *mrn1Δ* can be explained by the dysregulated expression of *NCA3*. *NCA3-tADH1* + *mrn1Δ* also grew more quickly than WT or *NCA3-tADH1* in the control cultures upon back-dilute into glucose-replete media (S5A and S5B Fig), which may indicate there is an initial fermentative growth advantage conferred by the upregulation of mitochondria and other cell wall proteins in the double mutant.

The mitochondrial proteome comprises roughly 5% of the cell’s total proteome during fermentation, and increases to 11% during diauxic shift, and up to 35% during ethanolic respiration [35]. At the same time, the small, spherical mitochondria seen during fermentative growth become elongated tubules during diauxic shift and ultimately form reticular networks of mitochondria during extended respiratory growth [35]. In light of our data linking Mrn1 to mitochondrial function, we were curious whether the *MRN1* deletion or the *NCA3-tADH1* mutation affected the shape and size of mitochondria. We visualized the mitochondria of log-phase, fermentative *NCA3-tADH1*, *mrn1Δ*, wild-type and *MRN1* overexpression cells using MitoTracker Red fluorescent dye (Fig 5D). Wild-type cells tended to have discrete mitochondrial punctae, with a small fraction of cells showing longer mitochondria. In *mrn1Δ* and *NCA3-tADH1*, mitochondrial staining was brighter and spread more broadly through the cell, and almost all cells had elongated mitochondria. In contrast, mitochondria in cells overexpressing *MRN1* were small and often difficult to visualize. We quantified these changes in mitochondrial volume using flow cytometric analysis of MitoTracker Red staining, which confirmed a 25% increase in *mrn1Δ* and *NCA3-tADH1* cells and a 25% decrease in *MRN1-OE* cells relative to wild-type (Fig 5E and 5F). These observations support our hypothesis that *mrn1Δ* adapts more quickly to respiratory growth due to increased mitochondrial function under fermentative growth, in part due to upregulation of *NCA3*, whereas *MRN1* overexpression inhibits mitochondrial expansion.

We also investigated how these mutations affected mitochondrial morphology during growth in non-fermentable media. Under these conditions, wild-type cells had expanded and elongated tubule-like mitochondria. Likewise, mitochondria in *mrn1Δ* cells were even larger and more abundant than those observed in fermentative growth and resembled the reticular-like morphology observed in wild-type cells following extended respiratory growth [35]. Cells overexpressing *MRN1* had more visible mitochondria with tubular morphology as well, which were similar albeit less abundant than the mitochondria we observed in wild-type cells. Surprisingly, the *NCA3-tADH1* cells had smaller and more spherical mitochondria than any of the other genotypes we examined (Fig 5G). This contrasts with the faster resumption of growth seen in *NCA3-tADH1* cells switched into non-fermentable media and the expanded mitochondria seen in fermentative growth (Fig 5C), but we note that we observed mitochondria 2 hours after shifting cells to respiratory conditions, whereas growth did not resume for 13 hours. Notably, *NCA3* was down-regulated in respiratory growth relative to fermentative conditions in both wild-type and *mrn1Δ*, whereas *NCA2*, another gene implicated in regulation of *ATP6* and *ATP8*, was upregulated. Interactions between these two genes regulating the mitochondria-encoded subunits of the F1-F₀ ATP synthase or other aspects of mitochondrial function may explain this surprising result.

## Discussion

We uncovered a role for Mrn1 as a regulatory RNA-binding protein with genetic and functional ties to both the mitochondrion and the cell wall. The unstructured N-terminus of Mrn1 triggered the degradation of bound RNAs, acting through *LSM3*. Full-length Mrn1, containing four RRMs, repressed a range of transcripts encoding proteins involved in cell wall biogenesis and mitochondrial oxidative phosphorylation. We found that loss of *MRN1* accelerated the cellular adaptation to respiratory growth, whereas Mrn1 over-expression inhibited this response. We observed a corresponding expansion of mitochondria in *mrn1Δ* cells, perhaps priming them for a shift to respiration, while Mrn1 over-expression cells had consistently smaller mitochondria. Mrn1-mediated degradation of the mitochondrial regulator *NCA3* contributes substantially to these effects.

**Fig 6:**
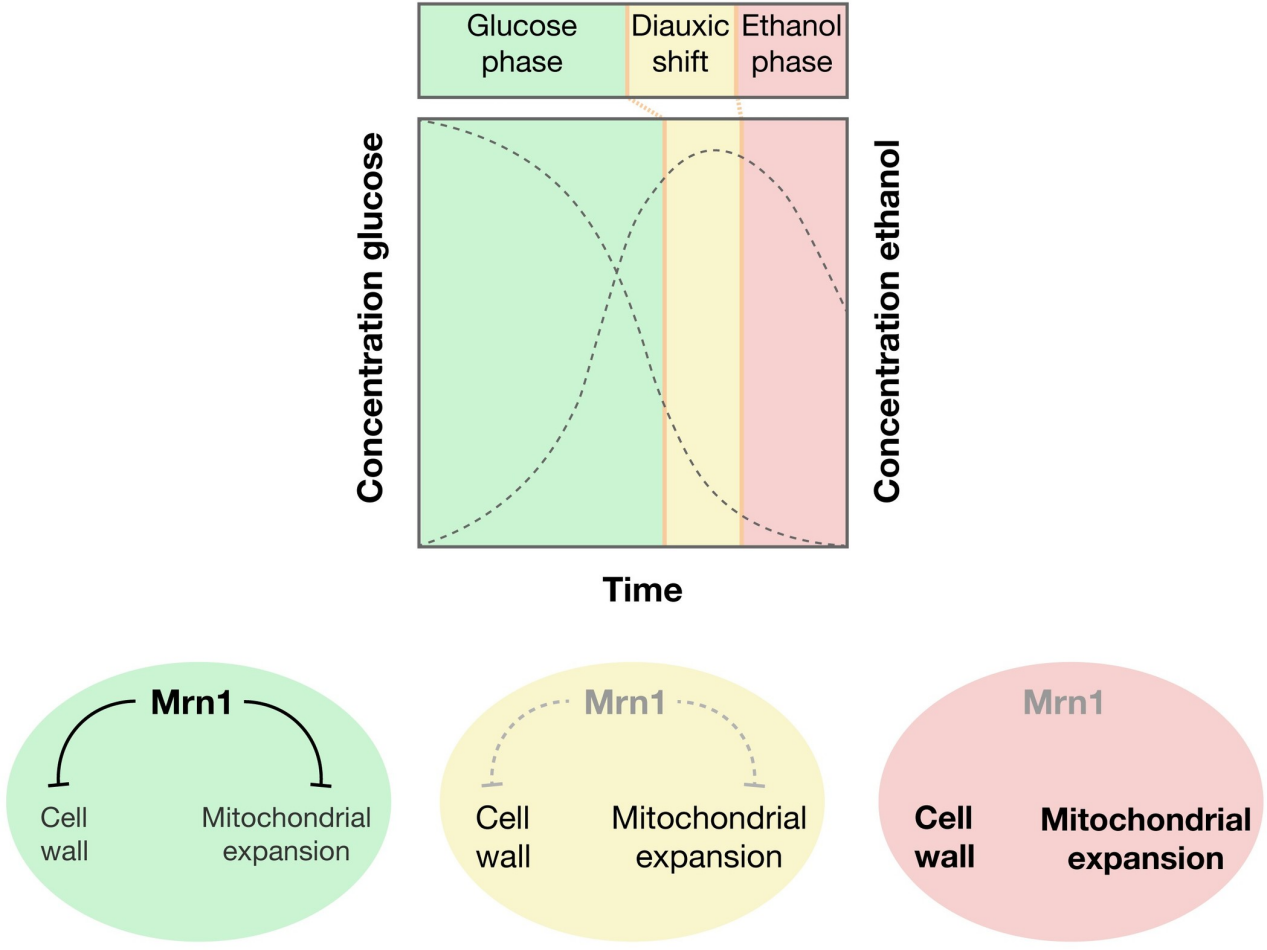
A model for the physiological role of Mrn1-mediated regulation. During fermentation, Mrn1 promotes the degradation of mRNAs involved in cell wall organization and mitochondrial expansion. Mrn1 is downregulated during the diauxic shift, and further repressed during long-term respiratory growth, allowing for the upregulation of proteins involved in cell wall organization and mitochondrial expansion.

Our results suggest an unappreciated link between cell wall biogenesis and mitochondrial function. We propose that Mrn1 acts as a nexus between these two physically distinct cellular compartments, mediating their responses to changing carbon sources (Fig 6). Mrn1 does this in part by regulating Nca3, and its paralogue Uth1, which are likewise linked to both organelles. We also found that Mrn1 interacts specifically with proteins that reside in both the mitochondrial and plasma membrane, although it is reported to localize diffusely throughout the cytoplasm during normal growth [9]. Other suggestive links have been reported between the mitochondrion and the post-transcriptional control of cell wall biogenesis. Most notably, RNA-binding protein Jsn1 physically and functionally associates with mitochondria [43], while Jsn1 and the paralogous Puf2 protein regulate *ZEO1* and thereby modulate cell wall stress responses [44]. While Mrn1 and Jsn1 control different transcripts, presumably for distinct purposes, they do highlight an important role for coordination of the cell wall and the mitochondrion.

Mitochondria produce ATP through oxidative phosphorylation during respiratory growth and carry out a range of other essential biosynthetic processes [45]. Production of the cell wall, which can comprise over a quarter of the cell’s dry weight, requires substantial energy along with carbohydrate biomass [31]. Thus, both organelles must respond to the availability of extracellular carbohydrates, and proper regulation of mitochondria has important implications for the cell. Mitochondria generate harmful free radicals as byproducts of the electron transport chain. These free radicals cause oxidative damage, which plays a major role in age-related degenerative diseases in higher eukaryotes [46]. Thus, the expansion of mitochondria that we observed in *mrn1Δ* cells, driven by inappropriate activation of its normal targets, could be deleterious. Indeed, the RNA-binding protein Puf3 regulates a distinct class of transcripts encoding mitochondrial functions, and loss of this regulation disrupts biosynthesis of the mitochondrial lipid, coenzyme Q [47].

Mrn1 is also enriched in interactions with proteins that localize to the mitochondrial matrix and inner membrane, relative to our cytoplasmic control (S4I and S4J Fig). Mrn1 is unlikely to interact with these proteins within the matrix, as it was not reported to show mitochondrial localization, and it lacks a predicted mitochondrial targeting sequence (MTS) [48]. However, Mrn1 could participate in the biogenesis or quality control of mitochondrial proteins in the cytosol. We saw interactions of Mrn1 with Tom22, a component of the translocase of the outer membrane (TOM) complex, and with Tim23 and Ssc1, which are essential components of the translocase of the inner mitochondrial membrane involved in protein folding and translocation (TIM23) [49–51] (S7 Dataset). Interestingly, CiBER-Seq indicated that knock-down of *TIM23* or *SSC1* both abrogated Mrn1 activity, as did knock-down of *SUE1*, a protein required for the degradation of unstable forms of cytochrome c [52] (S2 Dataset). These genetic interactions could indicate a role for Mrn1 in responding to protein translocation defects. Mitochondrial protein associated degradation, or mitoTAD, involves removal of precursor proteins from the TOM complex, and these stalled proteins often span the TOM and TIM channels [53]. Alternately, or additionally, Mrn1 may play a role in mitochondria-associated ribosome quality control (mitoRQC). This poorly characterized pathway involves recognition of stalled 60S ribosomes with nascent chains co-translationally inserted into the TOM complex [54]. Mrn1 could destabilize the mRNAs associated with these stalled complexes. However, it is difficult to distinguish these pathways based on the physical interactions of Mrn1. Finally, we note that Mrn1 may be bound to the outer surface of the mitochondrion and crosslink with topologically separated proteins only after lysis.

Mrn1 also interacted physically with plasma membrane proteins that are associated with the cell wall as well as mitochondrial function [55,56]. Notably, several of the proteins most-enriched in the Mrn1 fraction are also mRNA targets of Mrn1, including Pir1 and Ecm33, as well as various other cell-wall-regulatory proteins (S4K Fig). It is possible that Mrn1 interacts with these proteins in a co-translational manner while binding their cognate mRNAs, although Mrn1 was disenriched in proteins that localize to the ER. As in the case of mitochondrial proteins, Mrn1 could interact with these proteins during their normal biogenesis or in a quality control process. Notably, we also see attenuation of Mrn1 activity by certain guides expected to disrupt ER protein targeting and translocation (S2 Dataset).

The Mrn1 physical interactions together with the expression changes we observed confirm that Mrn1 links two physically distinct cellular compartments, the mitochondria and the cell wall, and coordinates mitochondrial responses to changing conditions. This coordinated regulation has broad implications for yeast cell growth and longevity.

## Materials and methods

### Strain construction

The dual reporter strain NIY293 was constructed by integrating pNTI282, encoding *pPGK1*::*YFP*::*BoxB*, into BY4741 at URA3 and pNTI473, encoding *pPGK1*::*mCherry*::*PP7*, into BY4742 at URA3 and mating the two strains together. The *mrn1Δ* strain was constructed by integrating a Kanamycin selection cassette into the endogenous MRN1 locus of BY4741, integration was confirmed via colony PCR. *Mrn1-OE*, *Mrn1(1–200)-OE* and *BFP-OE* strains were constructed by cloning an over-expression cassette of each gene driven by *pPGK1* expression into the EasyClone pCfB2189 vector, and integrating the linearized vectors into the XI-2 locus in BY4741. The strain NIY416 harbors a constitutively-expressed, genetically integrated copy of dCas9. We integrated the Kan-selectable vector pKS181 which expresses the ZEM synthetic transcription factor with a set of five repeat BoxB hairpins in the 3`UTR and a copy of eCitrine into the XII-2 locus of NIY416. We then integrated *Halo*::*LambdaN*::*3XFLAG* and *Mrn1(1–200)*::*LambdaN*::*3XFLAG* fusions into the XII-5 locus using the hygromycin-selectable pCfB2337 EasyClone vector. Plasmids for use in the tethering assay were constructed by cloning full-length *MRN1*, *MRN1(1–200)*, *MRN1(29–200)*, *MRN1(201–371)*, *MRN1(372–612)*, and Halo into the pKS137 vector which generates a LambdaN::1XFLAG::BFP fusion protein. Expression was confirmed through western blotting and selected for by gating for BFP-positive cells (S1C Fig). Guide RNA validation plasmids were generated by cloning gRNA oligos into pKS111, which encodes a gRNA scaffold driven by a *pRPR1* promoter and a *pGAL1*:: *mCherry* inducible expression cassette.

### Culturing conditions

Growth phenotype assessment via growth curve analysis was performed in triplicate cultures as follows: an overnight inoculum was prepared from a single clone from an agarose plate colony. The following morning, cultures were back-diluted to an OD600 of 0.1 in rich media with 2% glucose. At mid-exponential growth phase, or OD_600_ 0.6, cultures were either back-diluted back into rich media with 2% glucose as a control, or washed once in sterile water and resuspended in respiratory growth media containing 2% ethanol and 2% glycerol as the carbon source, or in rich media containing 0.6M NaCl. Growth was then monitored in a Tecan SPARK multimode plate reader at 30°C with shaking for up to 36 hours following back-dilution. For lithium acetate and heat shock analyses, *Mrn1(1–200)-OE*, full length *MRN1-OE*, and *BFP-OE* cultures were exposed to either 100 mM LiAc at room temperature for 20 minutes or stressed at 42°C for 20 minutes, then resuspended in rich medium and grown in the TECAN plate reader at 30°C for 24 hours with shaking. Triplicate cultures for flow cytometry were grown to mid-exponential growth phase in selective media and harvested at OD_600_ 0.6 by a 30-minute incubation in 4% paraformaldehyde.

### Library transformation

Yeast populations were transformed with plasmid libraries and maintained in SD-His media with 2% glucose at an optical density of OD_600_ 1.0 with 5 nM beta-estradiol to induce ZEM activation. When growth rate reached a steady state, pre-induction samples were collected prior to guide RNA induction with 250 ng/ml anhydrous tetracycline. Six doublings, or roughly 9 hours later, post-induction samples were collected, and then the cells were washed in sterile water then resuspended in SD-His with 2% ethanol and 2% glycerol and grown an additional three hours, roughly one doubling. Samples were then collected and prepared for high-throughput sequencing.

### Fluorescence measurement

Expression of YFP and RFP in the tethering assay was measured using a flow cytometric readout on a BD LSR Fortessa X20 with excitation by the 488mm blue laser and 561 mm yellow-green laser, captured on the FITC and PE-TexRed channels, respectively. Fluorescence measurements for 50,000 cells were collected for each sample, and gates were generated around 25% of the most actively-growing cells of the population.

### RNA quantification

Total RNA was harvested from triplicate cultures of each strain using the phenol chloroform method, as described in Nilsen, TW, 2013 [57]. Quantification of *YFP* reporter RNA expression in the tethering assay was performed via RT-qPCR analysis by comparing *YFP* Ct values to normalizer *RFP* Ct values, and experimental protein Ct values were compared to a tethered Halo control Ct values. *RAD51* and *NCA3* mRNA changes in *mrn1Δ* and *MRN1-OE* were calculated by comparing *RAD51* or *NCA3* expression to the housekeeping gene *UBC6*, and compared to expression by wild-type BY4741. rRNA depletion was performed using the QIAseq FastSelect yeast rRNA removal kit from Qiagen. cDNA was generated with Protoscript II reverse transcriptase from NEB, and cDNA end cleanup and adapter ligation was performed with the NEBnext Ultra II RNA Library Prep Kit with Illumina indexes. RNA sequences were quantified using single end sequencing technology with the Illumina HiSeq4000 sequencing platform.

### Barcode sequencing

All PCR reactions were performed using Q5 polymerase according to manufacturer protocols. DNA was purified using DNA clean & concentrator kits from Zymo, and when applicable AMPure XP beads were used to purify full-length DNA product. Size distributions and concentrations were measured before next generation sequencing using an Agilent TapeStation 2200.

### Sequencing data analysis

Sequencing data was processed using Cutadapt to remove sequencing adapter sequences and deconvolve multiplexed libraries based on embedded nucleotide indices. Trimmed barcodes were then counted and tabulated as described in [18]. Barcodes that lacked at least 32 counts in the pre-induction samples in one of the replicates were filtered out. The remaining barcodes were analyzed using DESeq2 analysis by comparing pre-induction Halo and Mrn1, and post-induction Halo as the pre-condition in our matrix to find significant genetic interactions in the post-induction Mrn1 sample [58].

### Protein expression analysis via Western blotting

Total protein was isolated from mid-exponentially growing yeast through rapid capture of protein expression through 5% tricarboxylic acid treatment for ten minutes, followed by a wash in acetonitrile. The cell pellets were then dried at room temperature for 30 minutes before bead-beating in cell lysis buffer for 5 minutes at room temperature. Samples were then resuspended in SDS-loading buffer from NuPage, boiled for five minutes, and loaded on 4–12% polyacrylamide Bis-Tris gels and separated by electrophoresis in MOPS buffer. Proteins were then transferred to a nitrocellulose membrane, and were blocked for 1 hour in TBST with 5% bovine serum albumin. Primary antibodies were incubated with membranes for one hour at room-temperature, washed with TBST, and then incubated for 30 minutes at room temperature with anti-rabbit and anti-mouse HRP-linked antibodies. Membranes were developed with Pierce ECL western blotting substrate and imaged on the chemiluminescence channel on a ProteinSimple.

### Proteomic analysis

Purifications were performed in biological triplicate for troubleshooting experiments and in single replicates for submission for LC-MS. Samples were collected from exponentially-growing cells through centrifugation, washing with ice cold buffer, and resuspension in lysis buffer containing 50 mM HEPES pH 7.5, 150 mM NaCl, and 1% Triton X-100 before flash-freezing in liquid nitrogen. Cells were lysed with cryogrinding by 6 cycles at 30 hertz for three minutes, the supernatant was clarified, and protein-protein interactions were cross-linked with 20 mM EDC chemical crosslinking reagent. The crosslinked lysate was then incubated with Halo Magna beads for 3 hours at 4°C. Samples were washed with buffer containing 8 M urea, 50 mM HEPES pH 7.5, 150 mM NaCl, and 1% Triton X-100. Samples were eluted from the Halo beads with TEV protease digestion and total eluates were prepared according to protocols from the UC Davis proteomics facility. Briefly, this entailed protein precipitation with TCA, 0.01M HCl 90% acetone washes three times, and air dried and resuspended in 100 mM ammonium bicarbonate. Samples were alkylated with 500 mM iodoacetamide and incubated in the dark for 30 minutes at 60°C. Proteins were digested in 100 mM ammonium bicarbonate and 4 ul of 0.5 μg/μl Trypsin. Tryptic digests were quenched with 50% formic acid, buffer-exchanged into 100 mM TEAB buffer, and the protein samples were labeled with four of the ten-plex labels from the Tandem-Mass Tag labeling kit by Thermo Fisher. Samples were speed-vacuumed to remove the supernatant and desiccate the proteins. Mass spectrometry analysis was performed at the UC Davis Proteomics Core Facility.

### Microscopy

Mitochondria of actively-growing cells were stained with 100 nM mitoTracker Red reagent at 30°C with shaking at 200 RPM for 30 minutes. Cells were washed twice with synthetic complete media, fixed in 4% paraformaldehyde for 15 minutes, and washed once with synthetic complete media then resuspended in 100 mM potassium phosphate buffer pH 7.0 with 1M sorbitol. Cells were immobilized on microscope slides with Prolong Gold antifade reagent with DAPI and incubated in the dark at room temperature for 24 hours. The mitochondria were then visualized using a Zeiss Z1 inverted microscope and images were captured with a Photometrics 95B sCMOS 1200x1200 camera on the 100X objective. Five fields of view were captured for each replicate of each strain, and approximately 5 representative cells were chosen for Fig 5 for each replicate.

## Supporting information

S1 Fig Flow cytometry measuring YFP (top) and RFP (bottom) absolute fluorescence units in the tethering assay with YFP-tethered (A) full length Mrn1, (B) Mrn1(1–200), (C) Mrn1(201–371), and (D) Mrn1(372–612) (n = 50,000 cells per sample, experiments performed in triplicate cultures). (E) Forward scatter absolute fluorescence units as a measure for cell size in the tethering assay (n = 50,000 cells per sample, experiments performed in triplicate cultures, one representative replicate shown). (F) Flow cytometry measuring activity of Mrn1 truncations in the tethering assay, as in Fig 1A, during respiratory growth (n = 50,000 cells per sample, experiments performed in triplicate cultures, one representative replicate shown). (G) As in (F), during osmotic stress. (H) As in (F), during osmotic stress. (I) Immunoblotting measurement of tethering construct abundance. Each construct contained a FLAG epitope tag. Lower bands indicate partial cleavage of T2A sequence between FLAG-tagged Mrn1 tethering construct and downstream SpHis5 protein (n = 2).
(PDF)

S2 Fig (A) Induction of ZEM-responsive reporter by β-estradiol measured by flow cytometry (n = 50,000 cells, 2 replicates per β-estradiol concentration). (B) RT-qPCR measurement of expression of RFP mRNA with Pat1 tethered to 3`UTR of ZEM, with and without ZEM induction, relative to tethering of an inactive control. (C) Flow cytometry measurement of RFP expression with Pat1, or an inactive control protein (Bfp), tethered to 3`UTR of the ZEM transcription factor (n = 3). (D) Ratio of RFP reporter to YFP normalization control, with either Mrn1(1–200) or an inactive control tethered to ZEM, prior to guide RNA induction. (E) Comparison of RNA barcode abundance for inactive Halo-tag tethering control, prior to guide RNA induction (*r* = 0.90). (F) As in (E), for Mrn1(1–200) tethering (*r* = 0.96). (G) As in (E), after guide RNA induction (*r* = 0.96). (H) As in (F), after guide RNA induction (*r* = 0.95). Error bars reflect standard deviation.
(PDF)

S3 Fig (A) Comparison of RNA-seq read counts in wild-type yeast in log-phase fermentative growth, replicate 1 versus replicate 2 (*r* = 0.98). (B) As in (A), comparing replicate 1 versus replicate 3 (*r* = 0.98). (C) As in (A), comparing replicate 2 versus replicate 3 (*r* = 0.99). (D) As in (A) for *mrn1Δ* yeast comparing replicate 2 versus replicate 3 (*r* = 0.99). (E) As in (D) comparing replicate 1 versus replicate 3 (*r* = 0.99). (F) As in (D) comparing replicate 1 versus replicate 2 (*r* = 0.99). (G) Growth of *mrn1Δ*, Mrn1 over-expression, and wild-type yeast during fermentative growth (n = 3, two representative replicates per strain depicted). (H) Growth of *mrn1Δ*, Mrn1 over-expression, and wild-type yeast during respiratory growth (n = 3, two representative replicates per strain depicted).
(PDF)

S4 Fig (A) Comparison of RNA-seq read counts in wild-type yeast switched into non-fermentable ethanol and glycerol media, replicate 1 versus replicate 2 (*r* = 0.98). (B) As in (A), comparing replicate 2 versus replicate 3 (*r* = 0.98). (C) As in (A), comparing replicate 1 versus replicate 3 (*r* = 0.99). (D) As in (A), for *mrn1Δ* yeast, comparing replicate 1 versus replicate 2 (*r* = 0.99). (E) As in (D), comparing replicate 1 versus replicate 3 (*r* = 0.99). (F) As in (E), comparing replicate 1 versus replicate 3 (*r* = 0.99). (G) Schematic of tandem affinity tag on endogenous Mrn1 and genomically-integrated mCherry. Streptavidin-binding peptide (Sbp), TEV protease cleavage site (TEV), and Halo-tag (Halo) are shown. (H) Immunoblot of Mrn1 and mCherry purified by Halo-tag capture on Halo resin and visualized by ɑ-Sbp staining (n = 3). (I) Comparison of Mrn1-interacting protein capture in respiratory and fermentative growth conditions. (J) Gene ontology analysis of proteins enriched in Mrn1 affinity capture relative to mCherry control. (K) Comparison of Mrn1 affinity capture with Mrn1 RNA binding score. mRNA’s with a binding score greater than Log2(1.45) above dotted grey horizontal line are considered Mrn1 targets [5]. (L) Comparison of Mrn1 affinity capture during fermentative growth with Mrn1 CiBER-Seq profile. (M) Comparison of Mrn1 affinity capture during respiratory growth with Mrn1 CiBER-Seq profile.
(PDF)

S5 Fig (A) Growth of *NCA3-tADH1+ mrn1Δ*, *NCA3-tADH1* and wild-type yeast in fermentable media: complete 20-hour growth curve and (B) first 8 hours after back-dilution (n = 3, two representative replicates per strain depicted in growth curve). (C) Flow cytometric analysis of MTR fluorescence as a measure of mitochondrial abundance. (D) Quantification of median MTR fluorescence in (C). Error bars reflect standard deviation (n = 2).
(PDF)

S1 Table Cellular compartment GO terms enriched in Mrn1 versus control. Gene names in bold indicate genes that both encode fungal cell wall proteins and also localize to or have a regulatory effect on the mitochondria.
(DOCX)

S1 Dataset Pre- versus post-gRNA induction CiBERseq data. (CSV)

S2 Dataset Post-gRNA induction Mrn1 CiBERseq data. (CSV)

S3 Dataset Wild-type pre- versus post-diauxic shift RNAseq data. (CSV)

S4 Dataset *mrn1Δ* pre- versus post-diauxic shift RNAseq data. (CSV)

S5 Dataset *mrn1Δ* versus wild-type pre-diauxic shift RNAseq data. (CSV)

S6 Dataset *mrn1Δ* versus wild-type post-diauxic shift RNAseq data. (CSV)

S7 Dataset Mrn1 protein-protein interactions proteomics data. (XLSX)
