# Supplementary figures and images for: Dynamic post-transcriptional regulation by Mrn1 links cell wall homeostasis to mitochondrial structure and function
Source: PLoS Genet. 2021 Apr 15;17(4):e1009521. doi: 10.1371/journal.pgen.1009521 (PMC8079021; doi:10.1371/journal.pgen.1009521)

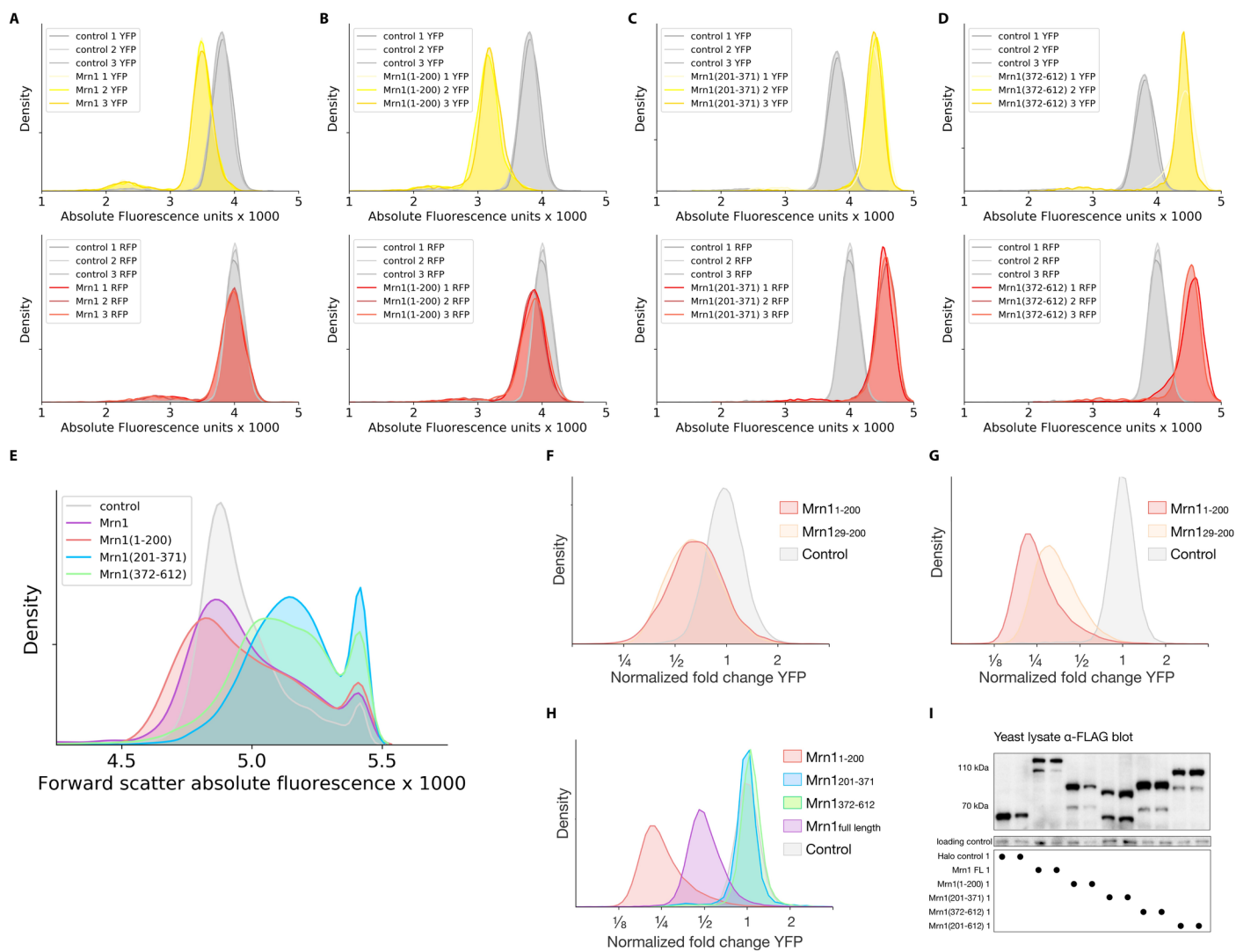

Supplement: S1 Fig — Flow cytometry measuring YFP (top) and RFP (bottom) absolute fluorescence units in the tethering assay with YFP-tethered (A) full length Mrn1, (B) Mrn1(1–200), (C) Mrn1(201–371), and (D) Mrn1(372–612) (n = 50,000 cells per sample, experiments performed in triplicate cultures). (E) Forward scatter absolute fluorescence units as a measure for cell size in the tethering assay (n = 50,000 cells per sample, experiments performed in triplicate cultures, one representative replicate shown). (F) Flow cytometry measuring activity of Mrn1 truncations in the tethering assay, as in Fig 1A, during respiratory growth (n = 50,000 cells per sample, experiments performed in triplicate cultures, one representative replicate shown). (G) As in (F), during osmotic stress. (H) As in (F), during osmotic stress. (I) Immunoblotting measurement of tethering construct abundance. Each construct contained a FLAG epitope tag. Lower bands indicate partial cleavage of T2A sequence between FLAG-tagged Mrn1 tethering construct and downstream SpHis5 protein (n = 2). (PDF) [file pgen.1009521.s001.pdf]

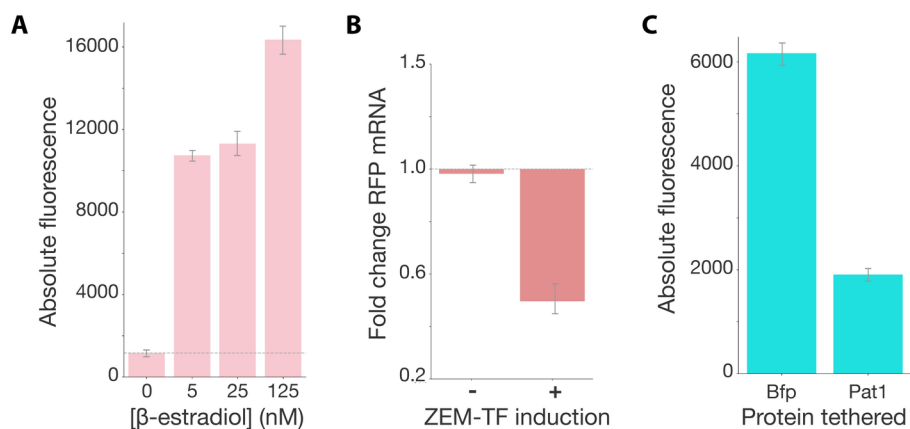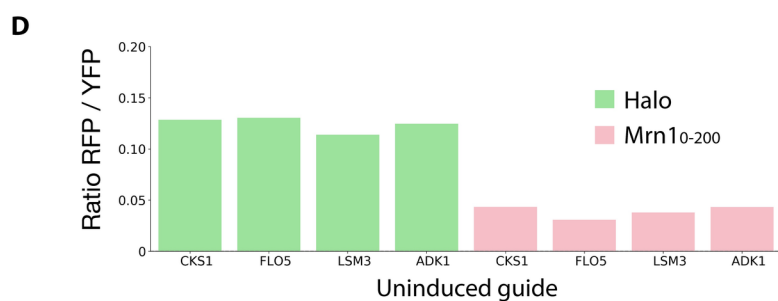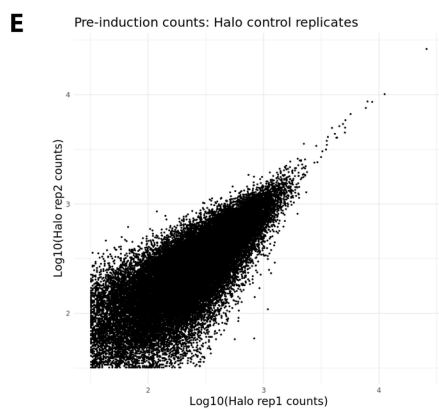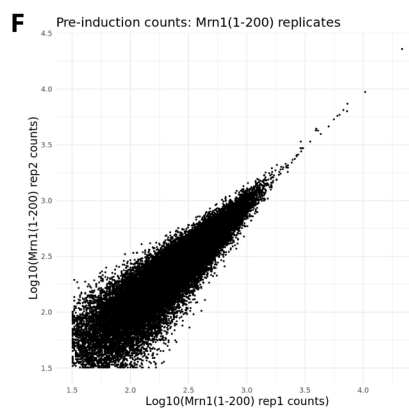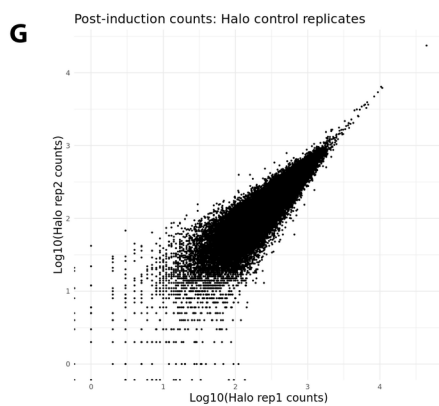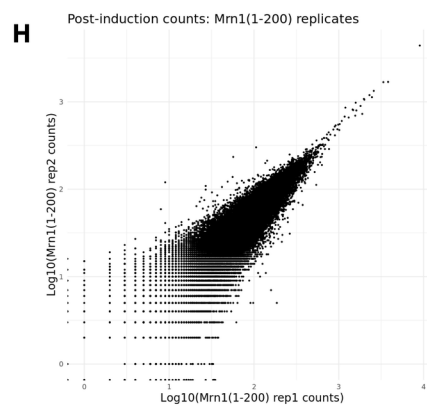

Supplement: S2 Fig — (A) Induction of ZEM-responsive reporter by β-estradiol measured by flow cytometry (n = 50,000 cells, 2 replicates per β-estradiol concentration). (B) RT-qPCR measurement of expression of RFP mRNA with Pat1 tethered to 3`UTR of ZEM, with and without ZEM induction, relative to tethering of an inactive control. (C) Flow cytometry measurement of RFP expression with Pat1, or an inactive control protein (Bfp), tethered to 3`UTR of the ZEM transcription factor (n = 3). (D) Ratio of RFP reporter to YFP normalization control, with either Mrn1(1–200) or an inactive control tethered to ZEM, prior to guide RNA induction. (E) Comparison of RNA barcode abundance for inactive Halo-tag tethering control, prior to guide RNA induction (r = 0.90). (F) As in (E), for Mrn1(1–200) tethering (r = 0.96). (G) As in (E), after guide RNA induction (r = 0.96). (H) As in (F), after guide RNA induction (r = 0.95). Error bars reflect standard deviation. (PDF) [file pgen.1009521.s002.pdf]

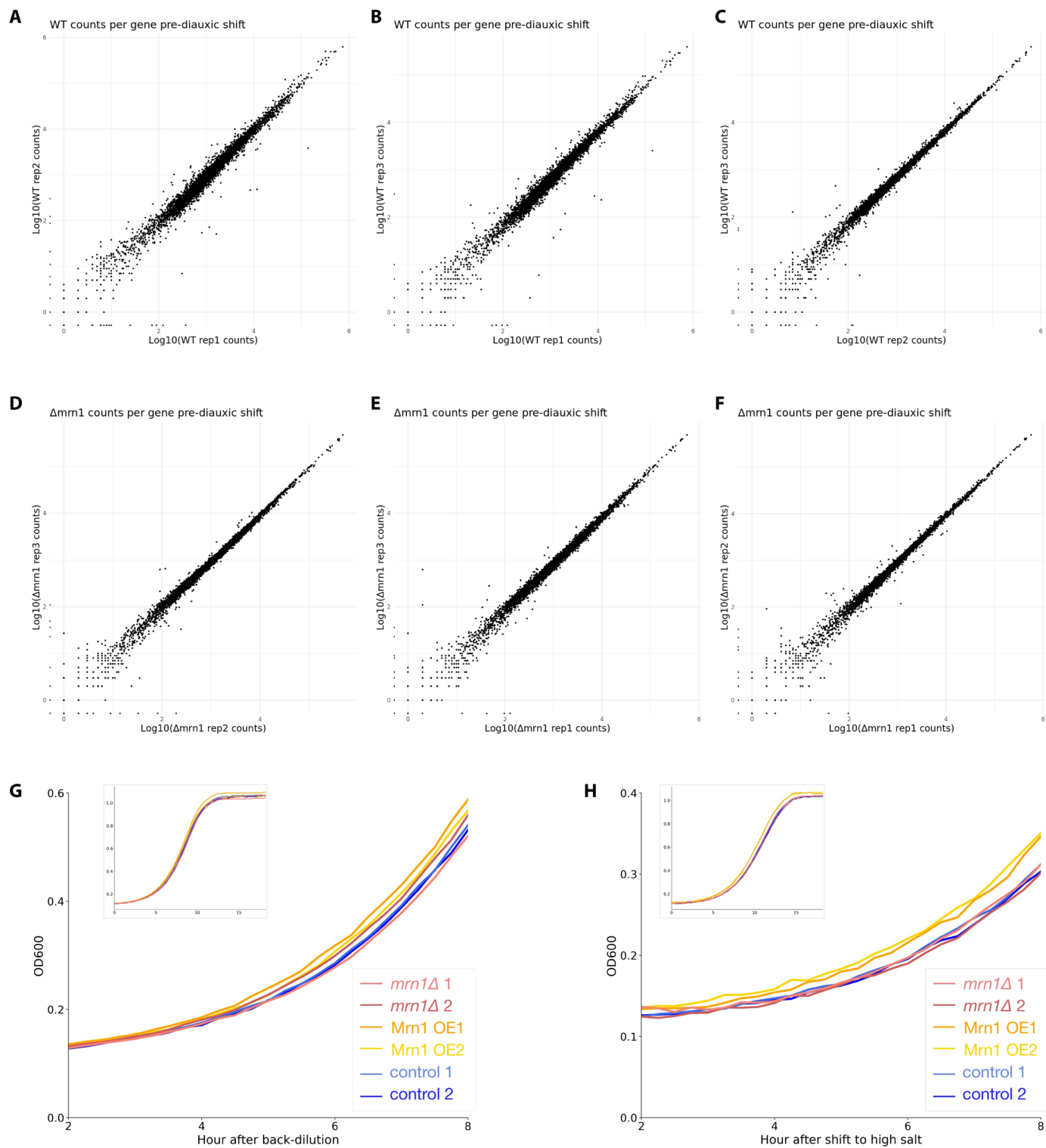

Supplement: S3 Fig — (A) Comparison of RNA-seq read counts in wild-type yeast in log-phase fermentative growth, replicate 1 versus replicate 2 (r = 0.98). (B) As in (A), comparing replicate 1 versus replicate 3 (r = 0.98). (C) As in (A), comparing replicate 2 versus replicate 3 (r = 0.99). (D) As in (A) for mrn1Δ yeast comparing replicate 2 versus replicate 3 (r = 0.99). (E) As in (D) comparing replicate 1 versus replicate 3 (r = 0.99). (F) As in (D) comparing replicate 1 versus replicate 2 (r = 0.99). (G) Growth of mrn1Δ, Mrn1 over-expression, and wild-type yeast during fermentative growth (n = 3, two representative replicates per strain depicted). (H) Growth of mrn1Δ, Mrn1 over-expression, and wild-type yeast during respiratory growth (n = 3, two representative replicates per strain depicted). (PDF) [file pgen.1009521.s003.pdf]

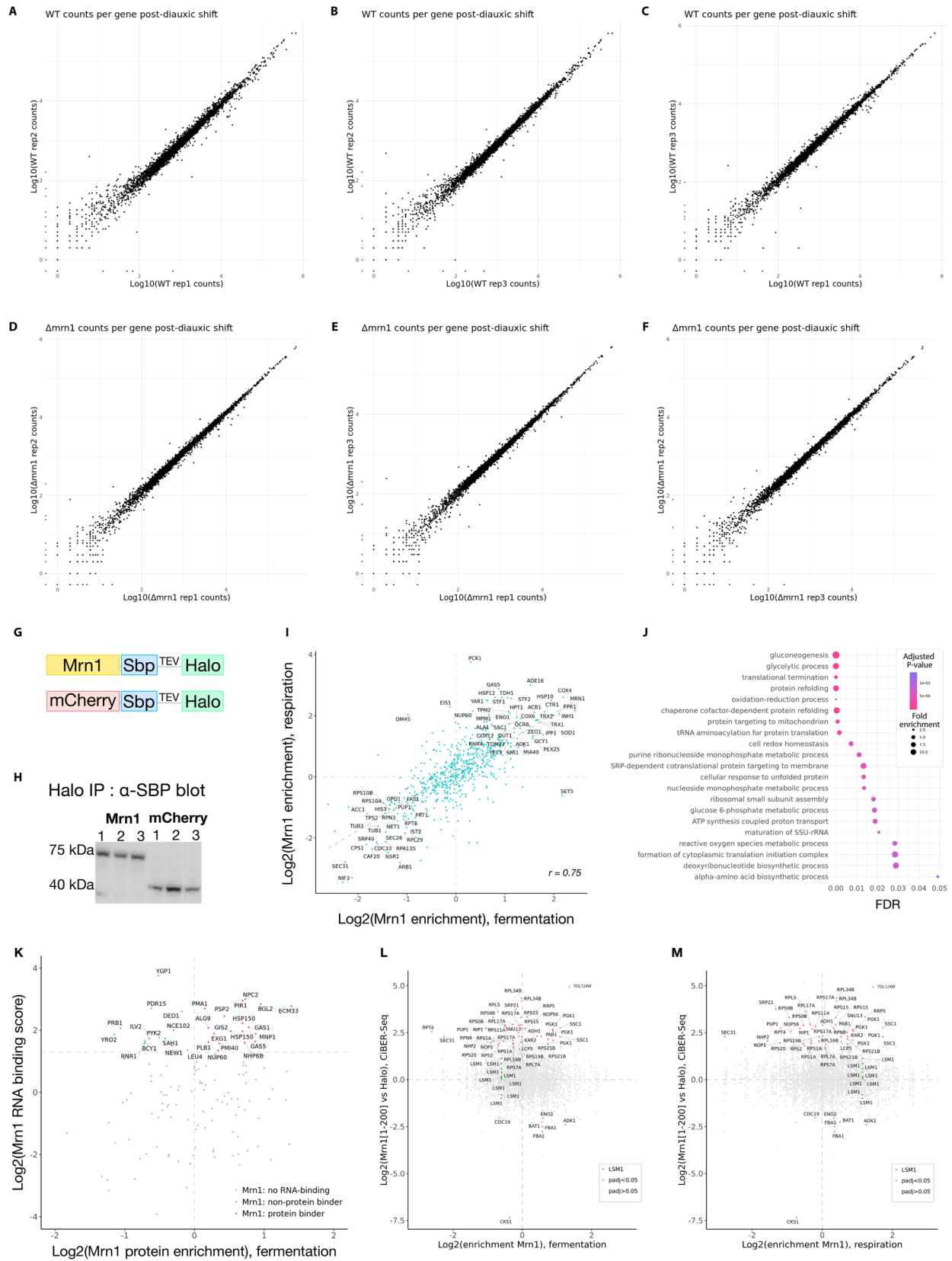

Supplement: S4 Fig — (A) Comparison of RNA-seq read counts in wild-type yeast switched into non-fermentable ethanol and glycerol media, replicate 1 versus replicate 2 (r = 0.98). (B) As in (A), comparing replicate 2 versus replicate 3 (r = 0.98). (C) As in (A), comparing replicate 1 versus replicate 3 (r = 0.99). (D) As in (A), for mrn1Δ yeast, comparing replicate 1 versus replicate 2 (r = 0.99). (E) As in (D), comparing replicate 1 versus replicate 3 (r = 0.99). (F) As in (E), comparing replicate 1 versus replicate 3 (r = 0.99). (G) Schematic of tandem affinity tag on endogenous Mrn1 and genomically-integrated mCherry. Streptavidin-binding peptide (Sbp), TEV protease cleavage site (TEV), and Halo-tag (Halo) are shown. (H) Immunoblot of Mrn1 and mCherry purified by Halo-tag capture on Halo resin and visualized by ɑ-Sbp staining (n = 3). (I) Comparison of Mrn1-interacting protein capture in respiratory and fermentative growth conditions. (J) Gene ontology analysis of proteins enriched in Mrn1 affinity capture relative to mCherry control. (K) Comparison of Mrn1 affinity capture with Mrn1 RNA binding score. mRNA’s with a binding score greater than Log2(1.45) above dotted grey horizontal line are considered Mrn1 targets [5]. (L) Comparison of Mrn1 affinity capture during fermentative growth with Mrn1 CiBER-Seq profile. (M) Comparison of Mrn1 affinity capture during respiratory growth with Mrn1 CiBER-Seq profile. (PDF) [file pgen.1009521.s004.pdf]

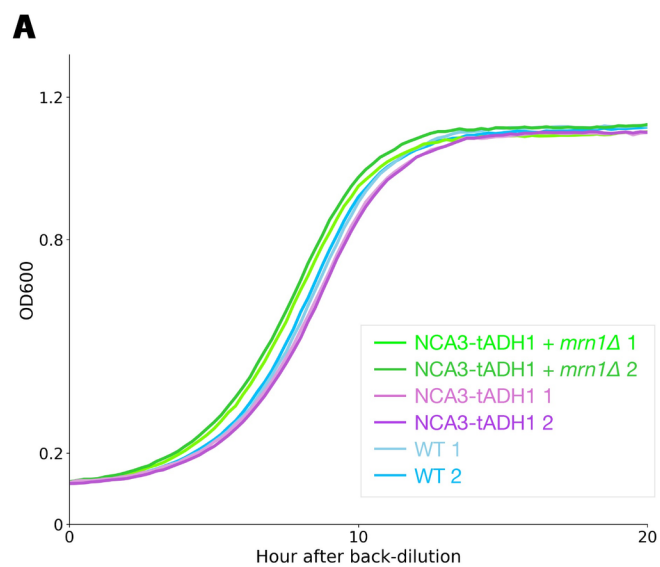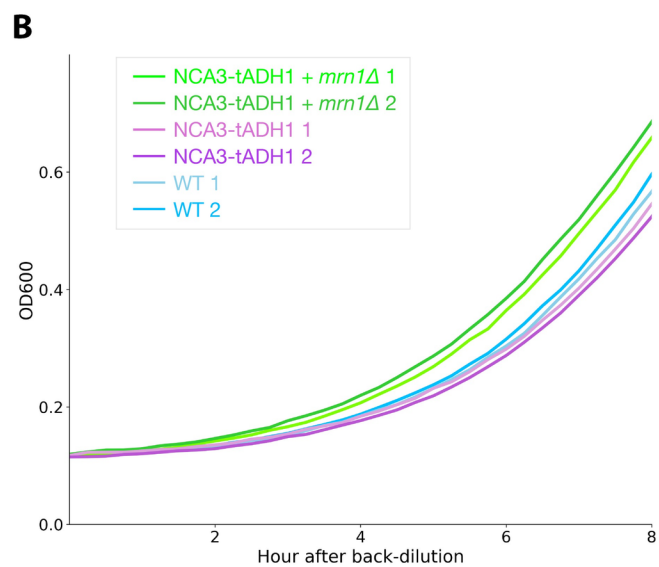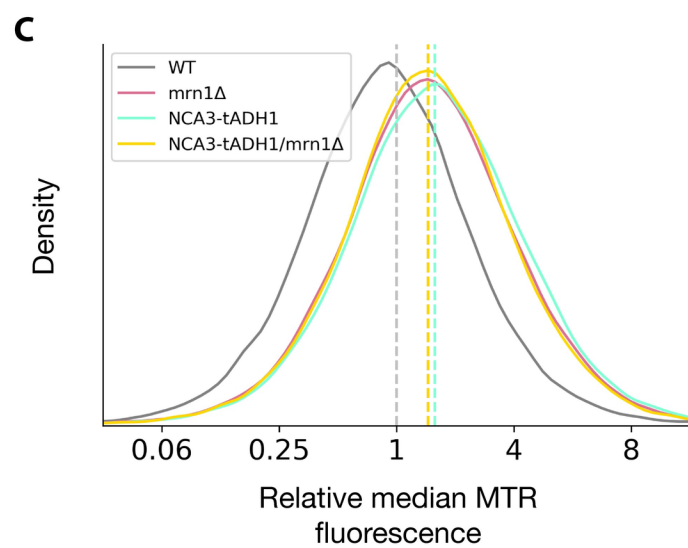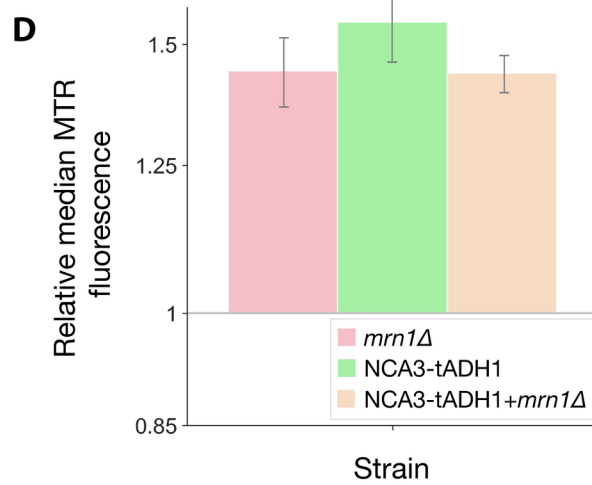

Supplement: S5 Fig — (A) Growth of NCA3-tADH1+ mrn1Δ, NCA3-tADH1 and wild-type yeast in fermentable media: complete 20-hour growth curve and (B) first 8 hours after back-dilution (n = 3, two representative replicates per strain depicted in growth curve). (C) Flow cytometric analysis of MTR fluorescence as a measure of mitochondrial abundance. (D) Quantification of median MTR fluorescence in (C). Error bars reflect standard deviation (n = 2). (PDF) [file pgen.1009521.s005.pdf]
