# Supplementary material for: Dynamic post-transcriptional regulation by Mrn1 links cell wall homeostasis to mitochondrial structure and function
Source: PLoS Genet. 2021 Apr 15;17(4):e1009521. doi: 10.1371/journal.pgen.1009521 (PMC8079021; doi:10.1371/journal.pgen.1009521)
Supplement: S1 Table — Gene names in bold indicate genes that both encode fungal cell wall proteins and also localize to or have a regulatory effect on the mitochondria. (DOCX) [file pgen.1009521.s006.docx]

**S1 Table. Cellular compartment GO terms enriched in Mrn1 versus control**

| **Cellular component GO Term** | **Proteins enriched** |
| --- | --- |
| Cytoplasmic stress granule | TIF11, ARC1, RPG1, PAB1, HSP26, TIF4631, YHB1, SES1, PBP1, HCR1, SBP1, HRP1, TMA19, TIF3, CYS4, BHM1, TIF2, FUN12, SUP35, MRN1, GIS2 |
| Translation preinitiation | TIF11, RPG1, HCR1, SUI3, FUN12 |
| Polysome | GIS2, SSA1, SSA2, TSA1, PBP1, BFR1, EGD1, SSE1 |
| Mitochondrial inner membrane space | COX4, CPR1, MIX17, HSP60, TRX1, TIM9, CYT1, MIA40, POR1, ADK1, RIB3, TIM8, TIM10, TIM13, ATP2, GPM1, QCR6, ACO1, SOD1, COX12 |
| Fungal cell wall | **PIR1**, **CTR1**, GAS1, CDC3, SSA2, CWP1, PIR3, TDH2, GAS5, CIS3, PHO3, EXG1, SSA1, TDH1, **ECM33**, PST1, CRH2, BGL2, TDH3, PIR5, **FBA1**, HSP150 |
